# Self-homodimerization of an actinoporin by disulfide bridging reveals implications for their structure and pore formation

**DOI:** 10.1038/s41598-018-24688-2

**Published:** 2018-04-26

**Authors:** Aisel Valle, Luis Benito Pérez-Socas, Liem Canet, Yadira de la Patria Hervis, German de Armas-Guitart, Diogo Martins-de-Sa, Jônatas Cunha Barbosa Lima, Adolfo Carlos Barros Souza, João Alexandre Ribeiro Gonçalves Barbosa, Sonia Maria de Freitas, Isabel Fabiola Pazos

**Affiliations:** 1 Laboratory of Molecular Biophysics, Institute of Biological Sciences, University of Brasília (IB-CEL/UnB), Campus Darcy Ribeiro, Asa Norte, Brasília DF-70910-900 Brazil; 2 Biochemistry Department and Center for Protein Studies, Faculty of Biology, Havana, University (Bq-CEP/UH), Universidad de la Habana, Calle 25 No. 455, Plaza de la Revolución, La Habana, CP-10 400 Cuba

## Abstract

The Trp111 to Cys mutant of sticholysin I, an actinoporin from *Stichodactyla helianthus* sea anemone, forms a homodimer via a disulfide bridge. The purified dimer is 193 times less hemolytic than the monomer. Ultracentrifugation, dynamic light scattering and size-exclusion chromatography demonstrate that monomers and dimers are the only independent oligomeric states encountered. Indeed, circular dichroism and fluorescence spectroscopies showed that Trp/Tyr residues participate in homodimerization and that the dimer is less thermostable than the monomer. A homodimer three-dimensional model was constructed and indicates that Trp147/Tyr137 are at the homodimer interface. Spectroscopy results validated the 3D-model and assigned 85° to the disulfide bridge dihedral angle responsible for dimerization. The homodimer model suggests that alterations in the membrane/carbohydrate-binding sites in one of the monomers, as result of dimerization, could explain the decrease in the homodimer ability to form pores.

## Introduction

*Stichodactyla helianthus* is an abundant anemone in the Caribbean Sea and in 1979 citolytic activity due to transmembrane pore formation originated from toxins partially purified from the same organism was first demonstrated^1,2^. In 1988, the purification of four hemolytic polypeptides of 17–20 kDa were first reported^3^, and in 2001 were purified two monomeric small toxins (20 kDa) with pore-forming activity, named sticholysin I (StI) and sticholysin II (StII)^4^. StI and StII have been functionally and structurally characterized^4–6^ and both toxins belong to the actinoporin protein family due to their purification from the order *Actiniaria* and its ability to form pores in membranes^7^. Actinoporins show high affinity for sphingomyelin (SM)-containing membranes^5,8–13^ and are classified as α-type pore-forming proteins (PFP) based on their mode of membrane integration through α-helical elements^14^. The actinoporin structure presents a typical β-sandwich fold flanked on each side by one short α-helix, where the N-terminal helix α1 is amphipathic and participates in the formation of the pore walls^15–17^. These toxins lack of post-translational modifications and have been successfully expressed in *E*. *coli* by different authors^18^. In addition, five phosphorylcholine (POC)-binding sites formed by conserved residues within the actinoporin family were identified, suggesting a multivalence binding mode during interaction with membrane lipids^15,17^. Recently, it has been demonstrated that Fragaceatoxin C (FraC), an actinoporin from *Actinia fragacea*, employs one of these lipid-binding sites for recognizing carbohydrates^19^. Many other physicochemical and structural aspects of the actinoporins such as, soluble structures, phospholipids-binding sites, multistep pore-formation mechanism, experimental approaches and theoretical tools for structure–function relationship studies, and the pore models toroidal, 9-mer conical α-helical-bundle and 8-mer conical hybrid α-helical-bundle/lipid have been described and reviewed recently^14,18,20–24^.

Actinoporins have attracted the attention for their potential use in biomedicine due to their high cytolytic activity, in the nanomolar concentration range. These toxins have been used in the construction of immunotoxins through their conjugation to monoclonal antibodies recognizing tumor-associated or parasite antigens, for selectively destroying different types tumor cell lines^25–32^ and the parasite *Giardia duodenali*s^33^. Besides, the funnel-shaped geometry described for FraC nanopores have been used for detection and sequencing of single-stranded DNA (ssDNA) distinguishing between homopolymeric C, T, and A polynucleotides^34^.

Most of the actinoporins isolated from sea anemones are cysteine-less proteins and consequently lack intramolecular or intermolecular disulphide bonds^20,35^. However, introduction of Cys residues by site-specific mutagenesis has been a useful strategy for understanding actinoporins structure-function relationship and for producing thiol-linked conjugates with different purposes^32,36–40^. A single mutant of wild-type recombinant StI (rStI) at position 111 from Trp to Cys (W111C) was produced for the construction of proteinase-activated immunotoxins and the impact of this mutation on the toxin pore-forming capacity was evaluated^38^. This W111C mutant (StI W111C) still presents pore-forming activity in a nanomolar range, but it is 8-fold less active than rStI. Furthermore, 90% of the StI W111C monomers spontaneously dimerize through disulfide bridging after 24 hours^38^. Potentially, a single exposed Cys residue in a monomeric actinoporin could be sufficient to induce spontaneously self-dimerization by disulfide bridge formation as has been reported for different single Cys mutants^38,39,41,42^. Several dimers of actinoporin Cys-mutants stabilized by a disulfide bridge located in functional regions, such as StI W111C dimer, are inactive or have reduced lytic activity, but this activity is recovered under reducing conditions. Following this reasoning, the property of inactive mutant dimers to recover pore-forming ability under reducing conditions, such as those found in intracellular endossomes, can be applied as a novel strategy for the delivery of antigens and other molecules into the cytosol^43^. In such a system, inactive dimers co-encapsulated with antigens in vesicles would first be compartmentalized into the cytosol via the endosomal-lysosomal system. Acidification of endossomes by the increase of H^+^ ions and other mechanisms would then promote reduction of disulfide bridge, giving rise to pore-forming capable toxins and triggering the release of antigens into the cytosol. This antigens delivery mechanism could promote cross-presentation of antigens artificially by MHC-I and thereby to stimulate cytotoxic T cell (CD8+) responses, and modulate or improve the key elements of the immune system^44,45^.

At this moment, the two forms of StI W111C dimers, the reversible dimer formed by disulfide bridge^38^ and the irreversible dimer formed by cross-linking via homo-bifuncional bis(maleimide)-hexane reagent^46^, have only been partially purified by molecular exclusion chromatography. So far, the low degree of purity attained for the StI W111C dimer has prevented its structural and functional characterization, thus limiting the progress of a strategy for the delivery of antigens into the cytosol. Hence a simplified procedure for dimer purification is still necessary to fully characterize their structure and mode of action. Therefore, in order to provide new insights into the structure and pore-forming activity of the StI W111C dimer, we reported for the first time: (i) the effective purification of StI W111C dimer stabilized by disulfide bridge of a pore-forming protein from *S*. *helianthus* sea anemone; (ii) the hemolytic activity in human erythrocytes of the purified dimer; (iii) the structural properties of the dimer evaluated by multi-spectroscopic techniques; and iv) a three-dimensional (3D) model of the dimer structure constructed by combining molecular modelling, a high-resolution library of disulfide bridge rotamers and molecular dynamics simulation.

## Results

### Expression and purfication of StI W111C mutant

The stI w111c gene was expressed in *E*. *coli* (16–20% of bacterial total proteins) using the ZYB-5052 autoinduction broth according to the 20 kDa protein band in 12% SDS-PAGE (Fig. S1 in the Supporting Material). StI W111C mutant was purified as a peak at 11.7 ± 0.6 mS/cm with hemolytic activity (HA) similar to other sticholysin Cys mutants^39^ (Fig. S2A) and its homogeneity and high purity were verified by SDS-PAGE (Fig. S2B) and HPLC-RP (Fig. S3A), respectively. The StI W111C purification yield was 10–27 mg of pure protein per liter of culture broth. The purified StI W111C showed two electrophoretic protein bands with 20 and 40 kDa corresponding to the molecular weights (MW) expected for monomer (60%) and homodimer (40%), respectively (Fig. S1B). HPLC-RP analysis of dimer also showed two peaks corresponding to monomer and dimer, with retardation times of 24.8 min and 25.6 min, respectively (Fig. S3A).

### Molecular weight

Matrix-assisted laser desorption/ionization with time-of-flight mass spectrometry (MALDI-TOF) mass spectra with a mass/charge (m/z) range of 2000–50000 was acquired for StI W111C monomer/dimer mixture (Fig. S4A), and rStI protein (Fig. S4B). The mass spectrum shows two ions with 38595.144 and 12876.662 m/z ratios corresponding to dimer mono-charged [StIW111C + 1H]^1+^ and tri-charged [StIW111C + 3H]^3+^, respectively. Ions with 19317.722 m/z ratio (Fig. S4A) could correspond to both the dimer di-charged [StIW111C + 2H]^2+^ and monomer mono-charged [StIW111C + 1H]^1+^. In addition, ions with 9655.708 m/z ratio (Fig. S4A) could correspond to both dimer tetra-charged [StIW111C + 4H]^4+^ and monomer mono-charged [StIW111C + 1H]^1+^. Mass spectrum of rStI shows only three ions with 19413.365, 9703.695 and 6467.590 m/z ratios (Fig. S4B) corresponding to mono-charged [rStI + 1H]^1+^, di-charged [rStI + 2H]^2+^ and tri-charged [rStI + 3H]^3+^ molecules, respectively. The average MW for rStI, StI W111C monomer and dimer forms were 19406 Da, 19313 Da, and 38618 Da, calculated based on majority consensus, respectively (Table 1).

**Table 1:** Molecular biophysical parameters of StI W111C monomer and homodimer.

Parameters		rStI	StI W111C homodimer	StI W111C monomer
Extinction Coefficients	ε_280 nm (mL mg_^−1^_cm_^−1^_)_	2.3 ± 0.2	2.00 ± 0.04	n.d.
	ε_280 nm_ *_(mL mg_^−1^_cm_^−1^_)_	2.2 ± 0.2	1.78 ± 0.03	n.d.
SV-AUC	S_20,w_ (S)	2.1 ± 0.1	2.5 ± 0.6	1.6 ± 0.4
	stokes radius (nm)	2.92 ± 0.63	4.45 ± 0.27	2.97 ± 0.54
	peaks integration (%)	97 ± 2	92 ± 2	8 ± 2
	Mw (Da)	18 381 ± 195	38 044 ± 1 227	19 202 ± 182
MW	theoretical **(Da)	19 390.02	38 545.9	19 272.96
	SEC ***(Da)	1 051.2 ± 1.41	12 417.1 ± 46.67	3 280.35 ± 8.55
	MALDI-TOF (Da)	19 406.17 ± 6.83	38 618.35 ± 17.21	19 313.57 ± 5.87
DLS	molecular diameter size (nm)	4.6 ± 0.9	5.9 ± 0.2	4.2 ± 0.9
	MW (kDa)	23.2 ± 4.7	41.9 ± 2.7	19.7 ± 4.3
	Pd (%)	20	3.9	19

Statistics from experiments to determine the molar extintion coefficients (ε), molecular weight (MW), analytical ultracentrifugation parameters by sedimentation velocity (SV-AUC) and hydrodynamic diameter (molecular size) by dynamic light scattering (DLS) measurements.

^*^Determination of molar extinction coefficient from 210–350 nm absorbance spectral measuring with correction for scattering contributions to the absorbance reading at 280 nm. It was not possible to determine (n.d.) the extinction coefficient of the StIW111C monomer, since that 15 mM of 2ME, a reducing agent to guarantee the exclusive existence of monomer, interferes in the 280 nm readings and protein concentration determinations by Lowry method.

^**^Theoretical MW were estimated by ExPASy server (http://web.expasy.org/).

^***^SEC experimental MW were estimated by size-exclusion chromatography.

The DLS values in the table are the results of experimental determinations from two independent protein samples. Pd: Polydispersity.

**Figure 1:**
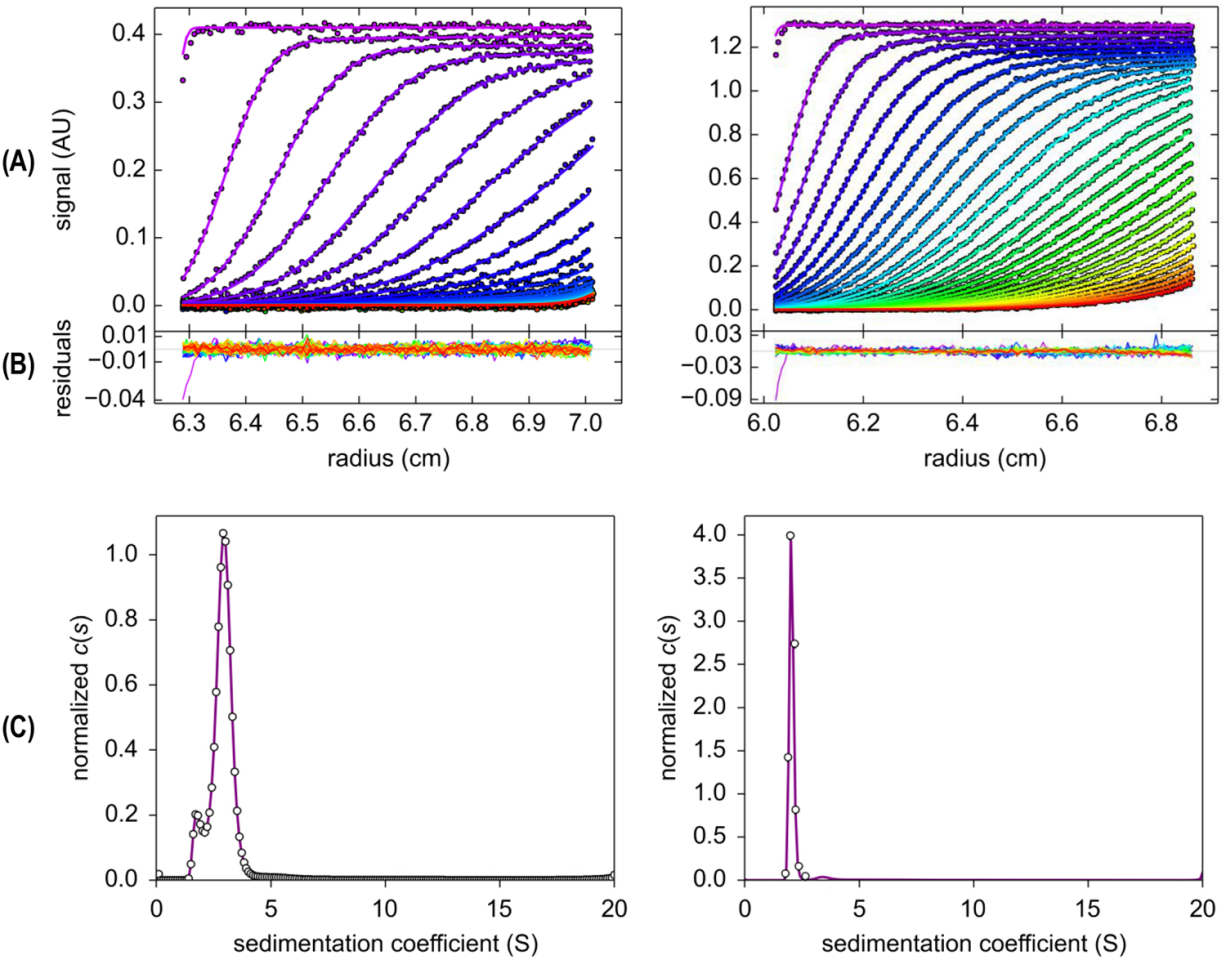
Sedimentation velocity analytical ultracentrifugation experiment. **(A)** Typical raw sedimentation profiles of absorbance at 280 nm versus cell radius for 24.4 mM StI W111C (left panels) and 24.7 mM rStI (right panels). The sedimentation scans were coloured with the progressive rainbow colours according to the software default setting: from violet, for those scans with the best fit and lowest residual, to those scans with the worst adjustment and the highest residual value. **(B)** Residual plot supplied by *SEDFIT* software showing the fitting goodness. **(C)** Continuous sedimentation coefficient distribution, c(S) curve, obtained with a regularization procedure from data shown in panels (A) and (B) with a confidence level of 0.90 using *SEDFIT* software and frictional ratio (f/fo) values between 1.136116 and 1.96157. The partial specific volume (*υ*) of the StI W111C and rStI monomers of 0.733957 and 0.734569 mL/g, respectively were determined using the *SEDENTERP* software. The density (ρ = 1.0182 g/mL) and the viscosity (η = 0.010415 poise) of TBS buffer solution were also determined by the *SEDENTERP* software. The symbols (open circles) represent experimental reading and the solid lines represent the best fitting to the *Lamm* equation supplied by *SEDFIT* software. Similar results were obtained for 12.25 mM of proteins concentration.

The values of the molecular weight of actinoporins previously determined by SEC chromatography using Superdex 75 were underestimated due to protein interactions with the resin^19^. Therefore, the StI W111C molecular weight and oligomeric states of the actinoporins were analyzed by velocity of sedimentation using analytical ultracentrifugation/ (SV-AUC). In the data analysis with *SEDFIT*, 176 scans of A_280 nm_ were included for each protein concentration (Fig. 1A) with good residual signals (Fig. 1B). The continuous sedimentation coefficient distributions curves, c(s), for StI W111 C (Fig. 1C, left) and rStI (Fig. 1C, right) were obtained. The rmsd values in the analyses of c(S) curves was 0.002, while the worst value was 0.01, suggesting that the model used was satisfactory. The MW, S_20,w_, and percentage contributions of the molecular species were determined by *SEDFIT* and *SEDPHAT* software. The c(s) curves for StI W111C showed two peaks (Fig. 1C, left) with S_20,w_ of 1.6 ± 0.4 S and 2.5 ± 0.6 S (Table 1). The corresponding MW and stokes radius values calculated for the first peak are 19 202 Da and 2.97 ± 0.54 nm, which could be associated to the monomer. The second peak with 38 044 Da and 4.45 ± 0.27 nm radius could be a homodimer formed by the association of two monomer linked by disulfide bridge (Table 1). For rStI only one peak (Fig. 1C, right) was observed and the average of relative S_20,w_ of 2.1 ± 0.1 S, MW of 18 381 Da, and stokes radius of 2.92 ± 0.63 nm were similar to the specie I of StI W111C (Table 1).

### Purification of StI W111C homodimers stabilized by disulfide bridge

**Figure 2:**
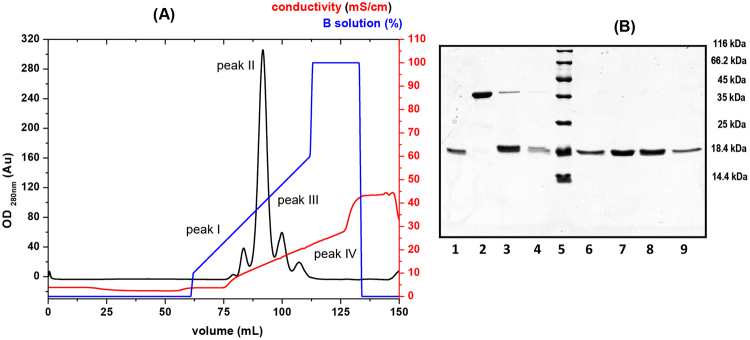
Purification of StIW111C homodimers stabilized by disulfide bridge. **(A)** Profile of ion-exchange chromatography of the StI W111C mutant on a *Sepharose-SP Fast Flow* column (r/L: 0.5/7.0 cm). The procedure was performed at 76.4 cm/h lineal flow and 1 mL fractions were collected on *ÄKTA primer* chromatography system. Purification was carried out from protein eluted from carboxymethyl cellulose (CM-52) column (Fig. S2). A_280 nm_ (OD280 nm) (black line), gradient of NaCl concentration in solution B (blue line) and conductivity (red line) are shown. **(B)** SDS-PAGE 15% of acrylamides^83^. Lanes 1, 2, 3 and 4: eluting fractions from peak I, II, III and IV without incubated with 2-mercaptoethanol (2-ME), respectively. Lanes 6, 7, 8 and 9: eluting fractions from peak I, II, III and IV incubated with 2-ME, respectively. Lane 5: Pierce unstained protein molecular weight marker (*Thermo Scientific*; USA).

In order to purifying dimer forms of StI W111C an ion-exchange chromatography using sulphopropyl (SP) groups as strong cation-exchanger (*Sepharose-SP Fast Flow* ion exchanger) was optimized and four elution peaks (I-VI) were observed (Fig. 2A). The SDS-PAGE analysis showed the presence of monomer in the peak I (Fig. 2B, lane 1) and only dimer form in the peak II (Fig. 2B, lane 2) with ~40 kDa of MW that reduces to monomer of ~20 kDa after incubation with 2ME (Fig. 2B, lane 7). This result confirmed the presence of only StI W111C homodimer stabilized by a covalent disulfide bridge in peak II. In the III and IV peaks, a majority presence of monomer and lower amount of dimer, produced probably from the dimerization spontaneous of this protein by disulfide bridge, were observed (Fig. 2B, lanes 3 and 4). Under reducing condition, only monomer of 20 kDa was detected in peaks I, II, and IV (Fig. 2B, lanes 6, 8 and 9). The monomers of the peaks I, III and IV elute at 9.9 ± 0.5 mS/cm; 13.4 ± 0.4 mS/cm; and 16.7 ± 0.5 mS/cm, respectively, suggesting that these species present different net or distribution charge probably due to conformational or chemical modifications. Most protein preparations, even those of equal purity, differ slightly in conformation and extent of modifications produced during extraction and purification processes, such as oxidation that includes Cys modification and affects the distribution of charges. The homodimer (peak II) elute at 20.0 ± 0.6 mS/cm and their purity was performed using HPLC-RP with retardation time 25.6 min and a high purity (>98%) (Fig. S2B).

**Figure 3:**
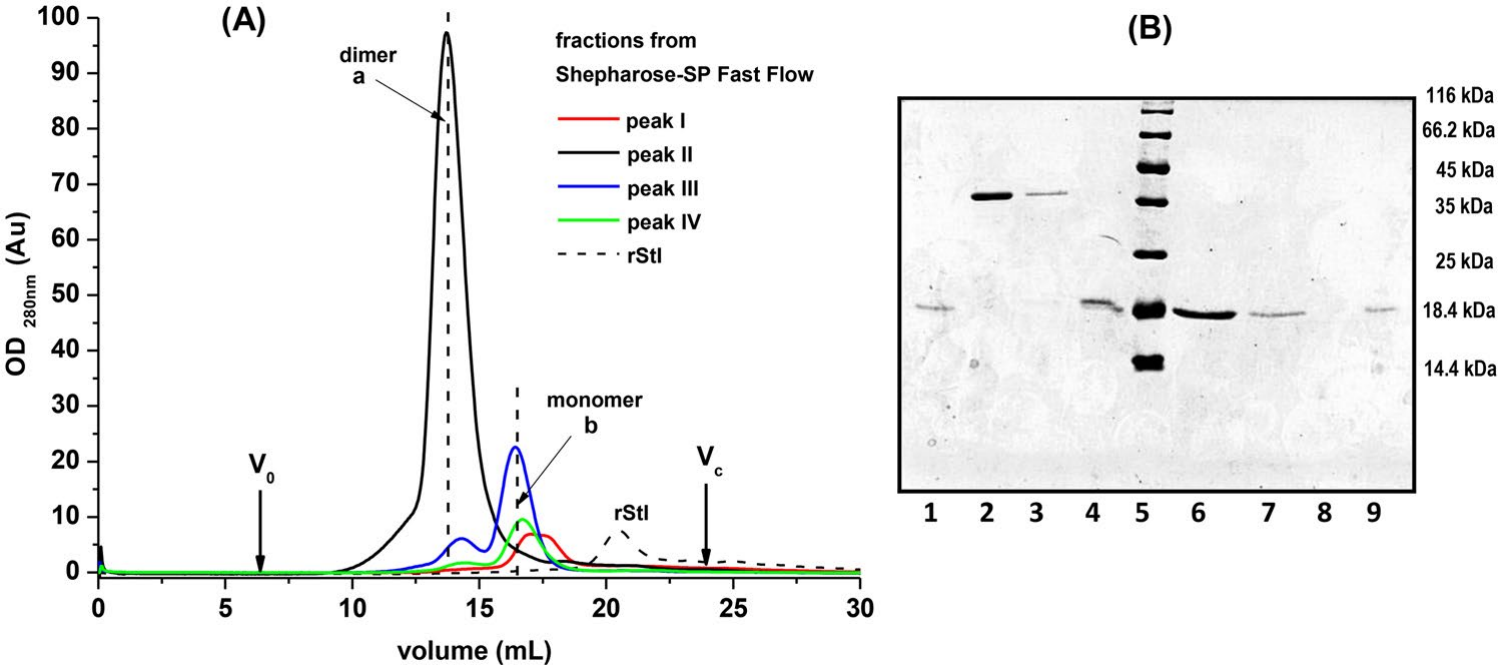
Molecular size-exclusion chromatography. **(A)** Analysis by size-exclusion chromatography (SEC) of StI W111C dimer and monomer forms eluted in fractions from *Sepharose-SP Fast Flow* cation-exchange chromatography (Fig. 2). Chromatography was carried out on a *Superdex 75 10/300 GL* (*General Electric*, USA) column with TBS buffer at a flow rate of 0.8 mL/min. The volume of protein injected was 1 mL from each peak, and the elution volumes for blue dextran 2000 (V_0_ = 6.95 mL) and the column bed volume (V_c_ = 24.04 mL) are indicated by arrows. The profiles of peak I (red line), II (black line), III (blue line) and IV (green line) from Sepharose chromatography are showed. The profile of rStI (black dashed-line) is indicated. MW of oligomeric form were estimated from calibration with a protein gel filtration molecular weight marker (*Low Molecular Weight* (LMW) *Kit* with range of 6 500 to 75 000 kDa). **(B)** SDS-PAGE 15% gel analysis^83^ of the size exclusion chromatography peaks. Lanes 1, 4 and 9 correspond to the fractions with monomers (peaks identified as b) from I, III and IV Sepharose peaks without incubate with 2-mercaptoethanol (2-ME), respectively. Lanes 2, 3 and 8 correspond to the fractions with dimers (peaks identified as a) from II, III and IV *Sepharose* peaks without incubated with 2-ME, respectively. Lanes 6, 7 and 9 correspond to the dimer forms (peaks identified as a) from II, III and IV Sepharose peaks incubated with 2-ME, respectively. Lane 5: Pierce unstained protein molecular weight marker (*Thermo Scientific*; USA).

The oligomers not stabilized by disulfide bridge of StI W111C, higher than dimers, are not stable during the SDS-PAGE and MALDI-TOF analysis, thereby their MW are not possible to determine using these procedures. In order to detect others oligomers in solution and to estimate their MW, SEC chromatography analysis of each elution peaks from SP cation-exchange chromatography fractions were carried out. The Fig. 3A shows the SEC elution chromatograms of the different molecular species present in each one four peaks (I–VI) obtained from *Sepharose-SP* (Fig. 2A). The elution volumes of peak I (17.3 ± 0.1 mL) and IV (16.7 ± 0.3 mL) were similar (peaks identified as *b* Fig. 3A) and containing proteins of 3280.35 ± 8.55 (Table 1) and 4157.7 ± 12.3Da, respectively, according to column calibration. However, the MW of these proteins is five times less than 20 kDa, as judged by SDS-PAGE analysis (see lanes 1 and 4, Fig. 3B). According to column calibration, the peak III presents proteins with MW of 10162.1 ± 38.89 Da (peak *a*, eluted at 14.3 ± 0.2 mL) and 4638.8 ± 13.86 Da (peak *b* eluted at 16.4 ± 0.3 mL) which were less than the analysis by SDS-PAGE, where they were characterized as a dimer (40 kDa, lane 3 Fig. 3B) and monomer (20 kDa, lane 4 Fig. 3B) species. The treatment of proteins from peak *a* with 2ME resulted only monomers (lane 7, Fig. 3B), suggesting that the StI W111C from peak III spontaneous homodimerized by a covalent disulfide bridge. The main peak of SP chromatography (peak II) (Fig. 2A) corresponds to homodimeric protein (40 kDa) according to SDS-PAGE (lane 2 Fig. 3B), but the MW according to the column calibration was less (12417.1 ± 46.67 Da) (Table 1) (peak *a*, eluted at 13.7 ± 0.3 mL, Fig. 3A). Treatment with 2ME generated only monomers of 20 kDa (see lane 6 Fig. 3B) confirming the dimerization by a covalent disulfide bridge. The rStI protein has a greater elution volume (20.4 ± 0.2 mL) than its mutant StI W111C in monomeric form (Fig. 3A) with a MW estimated by SEC of 1051.2 ± 1.41 Da (Table 1) that differs of 20 kDa by SDS-PAGE.

The distributions of the intensity as a function of particle diameter for a StI W111C dimer solution at 0.49 mg/mL (24.4 µM) in 0.02 M Tris-HCL pH 7.4 containing 140 mM NaCl buffer (TBS) at 20 °C showed one monodisperse peak (Fig. S5A) with hydrodynamic diameter and MW mean values around 5.9 nm and 41.9 kDa, respectively (Table 1). The polydispersity (Pd) of the peaks are equal or below 20%, indicating that the sample are monodispersity and homogeneity (Table 1). A dynamic light scattering (DLS) experiment was also performed under experimental conditions, using StI W111C monomer (obtained by incubation of dimers with 15 mM of 2ME) and rStI protein as a negative control of non-homodimerization by disulfide bridge (lacking Cys) (Table in Fig. S5). The results showed similar hydrodynamic diameters of 4.2 and 4.6 nm and MW values of 19.7 and 23.2 kDa for StI W111C monomer and rStI, respectively (Table 1).

### Molecular physicochemical parameters of StI W111C dimer

The molar extinction coefficients at 280 nm of StI W111C homodimers were estimated for using in the protein concentration determination. In order to determine the protein concentration with high precision the absorbance at 280 nm were corrected for the aggregates light scattering contribution with Eq. 1. The absorbance measures at 280 nm and determination of protein concentration by method of Lowry^47^ with BSA as standard using microplate reader was combined. The BSA standard curve showed a 620 nm linearity fit between 0.05–0.5 units for 0.1–0.8 mg/mL of protein with an extinction coefficient of 0.6 mL/(mg cm) similar to 0.66 mL/(mg cm) as reported (www.thermoscientific.com/pierce). The homodimer extinction coefficients were slightly different considering the light scattering contribution, 1.78 ± 0.03 mL/mg cm, and without light scattering correction, 2.00 ± 0.04 mL/mg cm (Table 1). In both determinations the coefficients of determination (Adj.R2) were 0.998. Since the light scattering influence in these results and is dependent on the MW, the radius of gyration, the size or shape of the aggregates^48^, and the protein concentration for the further characterization of dimer, were calculated from corrected light scattering spectrum.

### Hemolytic activity assays

**Figure 4:**
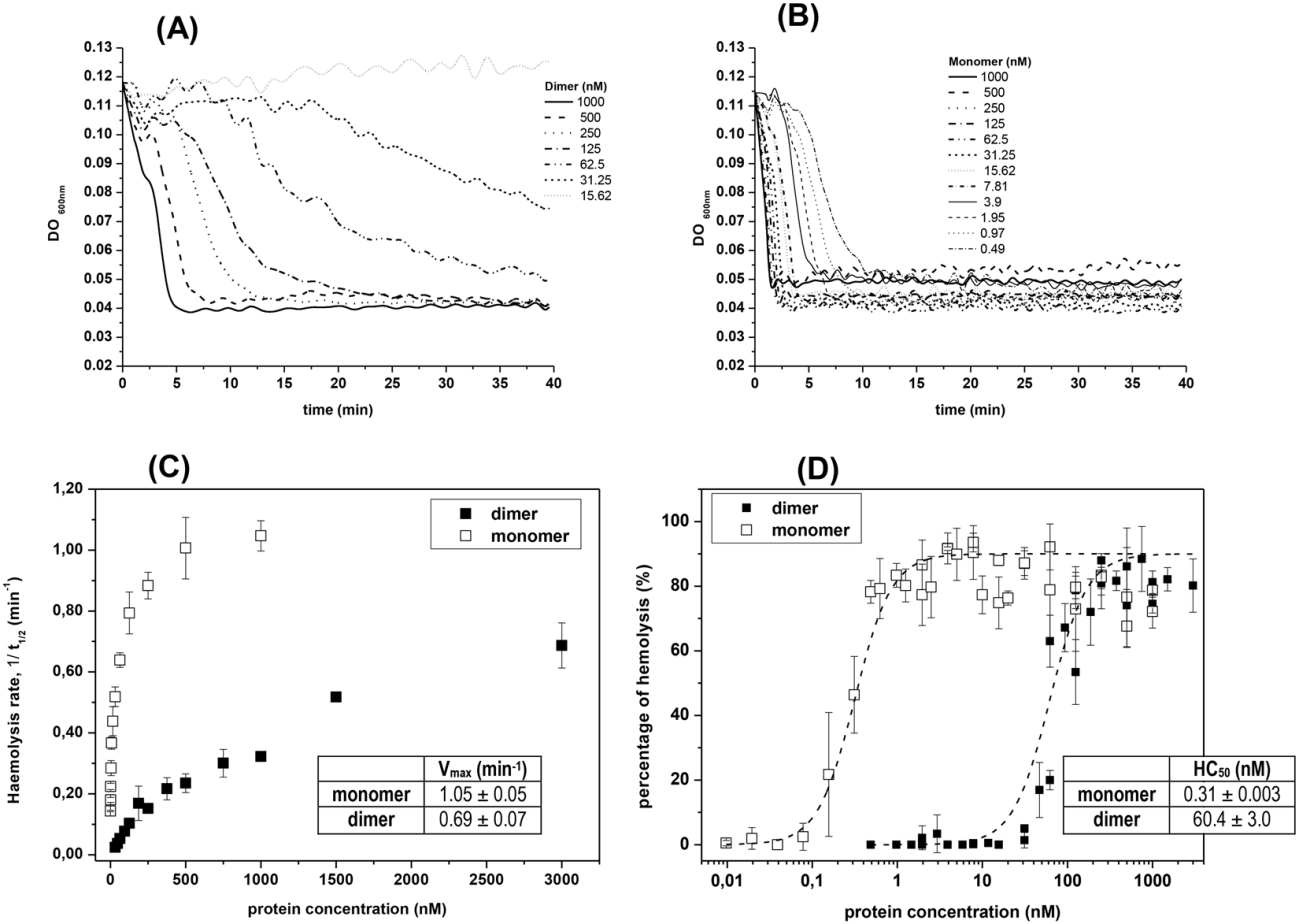
Hemolytic activity of monomer and dimer StI W111C forms. Time course of erythrocyte lysis of homodimer **(A)** and monomer **(B)** at different concentrations by measuring the decrease in the turbidity (DO_600 nm_) of an erythrocyte suspension. **(C)** dependence of the haemolytic rate respect to monomer and dimer concentrations. The kinetic parameters t_1/2_ (time when the initial DO_600 nm_ value of the erythrocyte suspension is reduced to the half) was estimated from curves on panels A and B. Rate of hemolysis is expressed as the reciprocal of the half-time of hemolysis (t_1/2_). **(D)** The percentage of hemolysis of dimer (without 2-ME) and monomer (dimer incubate with 0.1 M of 2-ME for 24 h) was determined for various doses of the toxin. The HC_50_ (concentration where 50% of HRBC are lysed after 15 min) of dimer (solid squares) and monomer (open squares) calculated from the best sigmoidal fit (dash lines) are shown in the inserted table. In panels C and D are shown the means and standard errors from 3–5 independent experiments.

Both StI W111C dimer and monomer showed lytic activity, but kinetics were markedly different. The hemolysis induced by dimer are characterized by a noticeable induction period for protein concentrations under of 250 nm followed by a relatively very-slow hemolysis (Fig. 4A). Induction periods are much smaller for monomer concentrations followed by very fast hemolysis rates (Fig. 4B). From the lysis curves the kinetic parameter t_1/2_ (time when the initial DO_600 nm_ value of the erythrocyte suspension is reduced to the half) for each oligomeric form at different toxin concentrations were estimated. The reciprocal values of the parameters t_1/2_ are directly proportional to the hemolytic rates of the reaction^49^. Hemolytic rate versus protein concentrations were exponential and theirs maximum velocity of lytic activity (V_max_) were estimated (Fig. 4C and inserted table). The V_max_ of the dimer (0.69 min^−1^) is 1.5 times lower than monomer (1.05 min^−1^), and was achieved at 3 uM and 1 uM for dimer and monomer, respectively (Fig. 4C and inserted table). The percentage of hemolysis of StI W111C monomer and dimer were also calculated with Eq. 2. An increase in hemolytic activity (HA) percentage as a function of protein concentration logarithm was observed (Fig. 4D) following a sigmoid pattern. Noticeable differences were observed between the ranges of concentrations needed to achieve a complete hemolysis by both oligomeric forms. The StI W111C dimer showed a higher protein-activity concentration range (20–1000 nm) than monomer (0.1–4 nm) (Fig. 4D), which is more active than dimer. For quantitative comparative purposes the parameter HC_50_ (protein concentration that lyses 50% of the erythrocytes) can be considered. The HC_50_ is approximately 0.31 nm and 62.4 nm for monomer and dimer, respectively (Fig. 4D and inserted table).

### Conformational characterization of StI W111C homodimers

The structural differences between monomers and homodimers were analyzed by CD spectroscopy. The monomers were obtained by incubation of the dimers with 15 mM of 2ME during 24 h and the disulfide bridge reduction was confirmed by SDS-PAGE. The Far-UV CD spectra and secondary structure contents of the rStI and StI W111C dimer (Fig. S6A) were very similar. The StI W111C monomer spectrum showed a slight decrease in the α-helices content with consequent increase in β-structures (Fig. S6A), probably due to 2ME contributions. As expected for actinoporins^39,50–52^ the CD spectra of the three proteins showed a positive band at 195–200 nm and a negative band at 217–218 nm^53^ (Fig. S6A) typical for proteins with a high content of β-sheet. The spectral features in the Near-UV CD region are largely similar for rStI, StI W111C monomer and dimer forms respect to positions of the aromatic-bands around 250–350 nm (Fig. S6B). The presence of Near-UV CD significant signals is a good indication that the proteins are folded into well-defined structures and the similarity in the band positions indicate a similar global structural folding of the proteins. The StI W111C monomer present a slightly increase of Trp intensity band (290–300 nm) respect to rStI as result to the W111 substitution by Cys (Fig. S6B). In contrast, the aromatic bands for StI W111C dimer are displaced towards more positive values (Fig. S6B). The observed displacement can be due to the contributions of the disulfide bridge in the StI W111C homodimer structure.

**Figure 5:**
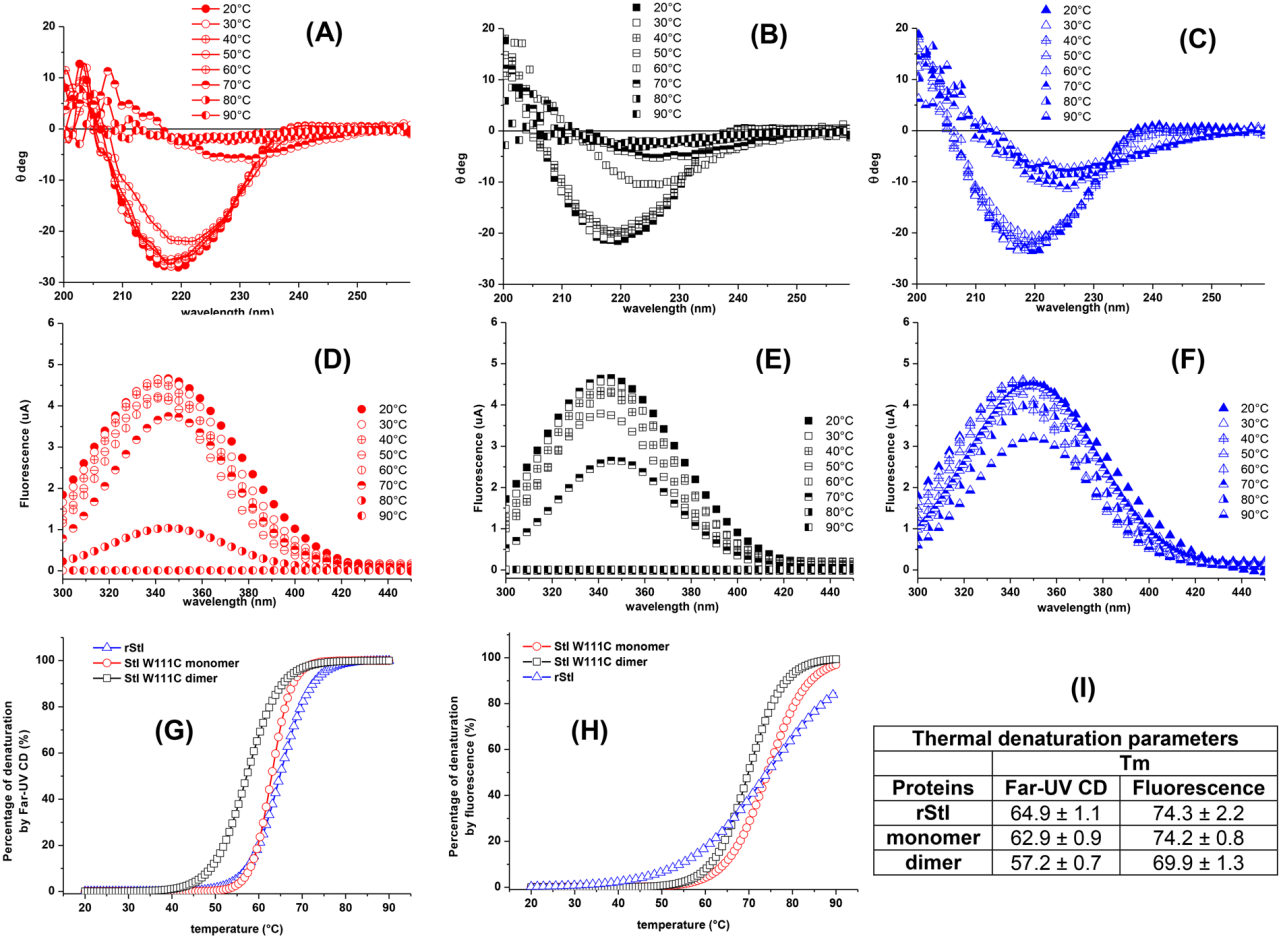
Thermal unfolding of rStI, StI W111C monomer and dimer forms by by Far-UV CD and fluorescence emission. Far-UV CD spectra of StI W111C monomer **(A)**, dimer forms **(B)** and rStI **(C)** were recorded in TBS buffer at 20–90 °C temperature range. Tryptophan fluorescence emission of StI W111C monomer **(D)**, dimer forms **(E)** and rStI **(F)** were measured at 334 nm, after excitation at 295 nm, in the same conditions. Protein concentration was 0.05 mg/mL. In each case, the baselines have been subtracted. Thermal dependence of percentage protein denaturation by Far-UV CD **(G)** and the tryptophan fluorescence emission **(H)** are depicted. The symbols are as follows: (circles) StI W111C monomer, (squares) dimer and (triangles) rStI. The treatments for the 20–90 °C temperature range are specified in the legend of panel. Melting temperatures (Tm) values **(I)** correspond to the temperature at the midpoint of the monophasic thermal transition calculated from percentage of change of ellipticity at 220 nm and fluorescence at 334 nm as function of temperature.

Additionally, structural changes and thermal stability of rStI, StI W111C monomer and dimer forms in solution were investigated by Far-UV CD. As shown, negative band at 218–220 nm have been a characteristic signature of predominant β-sheets structures in rStI and Cys mutants^39,42,54^. Therefore, the position changes of characteristic band of rStI, StI W111C monomer and dimer forms at 218–220 nm towards 225–230 nm and their intensity decrease as a function of temperature were observed (Fig. 5A–C). This distinctive feature indicates the gradual unordered structure formation. As shown in Fig. 5A and B, temperature increase above 50 °C results in a drastic decrease in ellipticity at 220 nm of StI W111C dimer and monomer indicating the whole-protein unfolding process. In contrast, the complete unfolding process of the rStI is not verified, as indicated by similar ellipticities at 220 nm as a function of temperature (Fig. 5C). Additionally, the Far-UV CD spectra of rStI, StI W111C monomer and dimer, recorded in the range 20–90 °C, do not present an isodichroic point^55,56^ (Fig. 5A–C).

Conformational changes of rStI, StI W111C monomer and dimer as a function of temperature were also investigated by Trp fluorescence emission at 334 nm after excited with 295 nm. When the temperature raising from 20 to 90 °C, the fluorescence values at 334 nm decreases more quickly for StI W111C dimer (Fig. 5E) followed by monomer form (Fig. 5D) and rStI (Fig. 5F). The denaturation percentage versus temperature was considered for quantify the unfolding process based on the ellipticity at 220 nm and fluorescence emission at 334 nm at 20 and 90 °C. Far-UV CD (Fig. 5G) and fluorescence emission (Fig. 5H) curves indicated that thermal denaturation of the three proteins is a cooperative two-state transition process. The melting temperature (Tm) was calculated as the temperature in which 50% of the total protein is in fold/unfolded states. In this case, the denaturation percentages estimated by ellipticity changes at 220 nm (Eq. 4) of rStI, StI W111C monomer and dimer do not depend on temperature in the range 20–45 °C and then drastically decreases over a narrow temperature range (Fig. 5G). The denaturation curves of StI W111C dimer and rStI have similar features (Fig. 5G), but different Tm centered at 57.2 °C and 64.9 °C, respectively (Fig. 5I). However, the StI W111C monomer and rStI curves show different features but equals Tm values (64 °C) (Fig. 5I). The fluorescence data indicate that rStI and StI W111C monomer are the most stable proteins, since they unfold at Tm 74 °C, whereas StI W111C dimer unfold at 69.9 °C (Fig. 5H,I). It is important to note that above 60 °C, changes in the secondary structure are drastic (Fig. 5G), whereas those in the tertiary structure occur above 70 °C (Fig. 5H).

**Figure 6:**
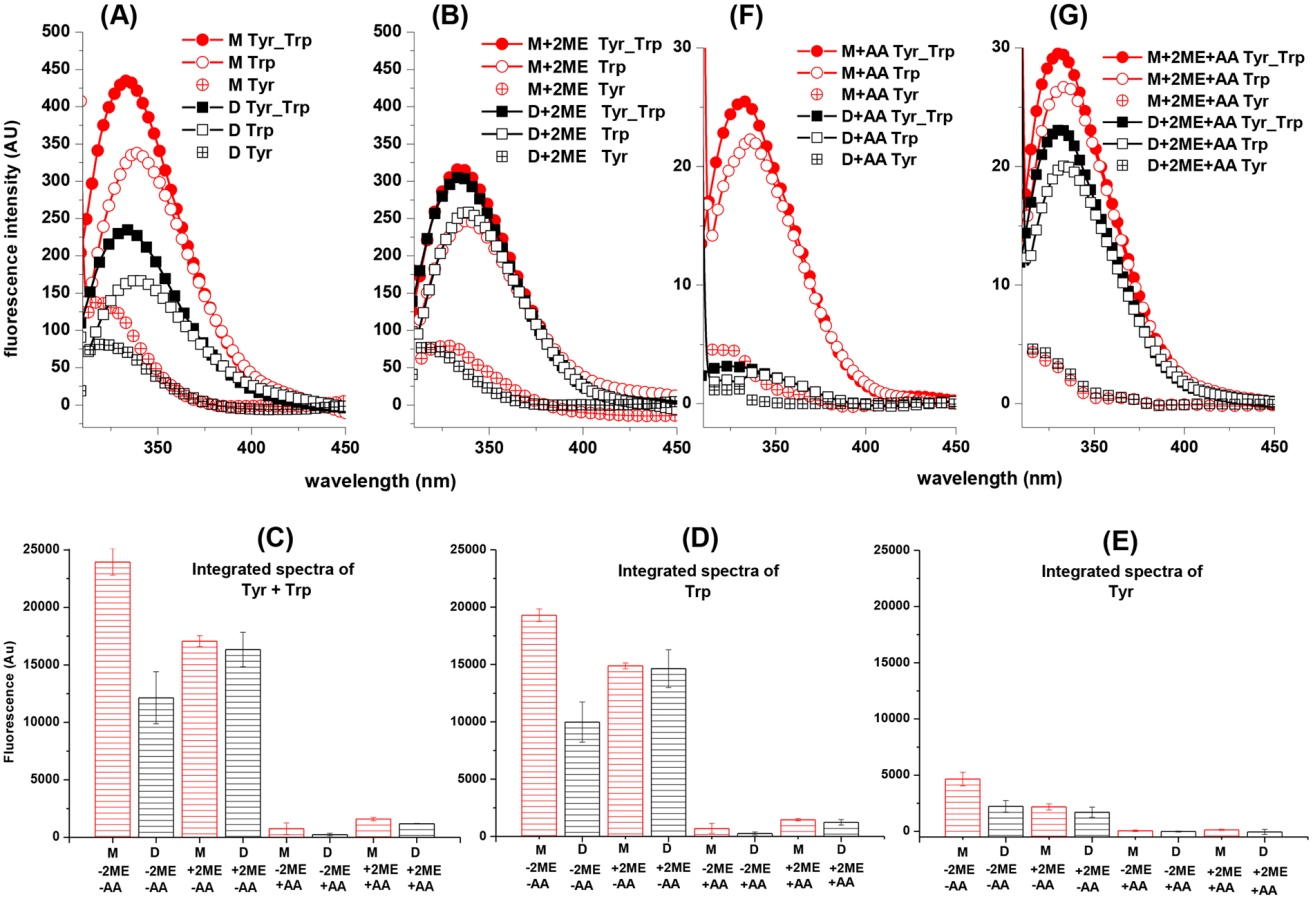
Fluorescence emission spectra of monomer and homodimer StI W111C forms. Fluorescence emission spectra of Trp and Tyr residues from monomer and dimer forms of StI W111C mutant in solution **(A)** and pre-incubating the sample with 15 mM 2-mercaptoethanol (2ME) during 24 h **(B)**. The spectra labeled with solid symbols resulted from excitation at 275 nm (Trp and Tyr contribution) and the spectra labeled with open symbols resulted from specific excitation of Trp at 295 nm (Trp contribution). Trp spectra were normalized multiplying each fluorescence intensities by the ratio between the emission intensities of Trp/Tyr and the Trp spectra at 380 nm, in which the emission of Tyr does not occur. Tyr spectra (open symbols and cross) were obtained by subtracting the Trp-normalized spectra and spectra of protein with excitation at 275 nm (Trp and Tyr contribution). Panels **(C**–**E)** show the integrated spectra values from Trp-Tyr, Trp and Tyr contributions, respectively. All spectra were recorded at 1 µM of protein concentrations and fluorescence emission is proportional in all cases but expressed in arbitrary units. The following labels, colors and symbols represent the different protein samples: monomer (M-2ME-AA, red, circle) and dimer (D-2ME-AA, black, square) forms in solution; monomer (M + 2ME-AA, red, circle) and dimer (D + 2ME-AA, black, square) forms in solution pre-incubated with 2ME; monomer (M-2ME + AA, red, circle) and dimer (D-2ME + AA, black, square) forms in solution quenched with 300 mM of acrylamide (AA); and monomer (M + 2ME + AA, red, circle) and dimer (D + 2ME + AA, black, square) forms in solution pre-incubated with 2ME and quenched with 300 mM of acrylamide (AA). Fluorescence maximum emission wavelengths (max) were calculated and shown in Table 2. (**F**) and (**G**) are equivalent to panels A and B but the experiments were carried out with 300 mM acrylamide (AA) as quencher.

Homodimer showed a fluorescence emission about 50% smaller respect to the monomer spectra of Trp, Tyr, and both residues (Fig. 6A), but the maximum emission-wavelength (λ_max_) values were similar for each type of residues (Table 2). In order to evaluate the fluorescence quenching of 2ME, the monomeric form present in peak I of SP chromatography (Fig. 2B, lane 1) was included in study. The Fig. 6B shows the fluorescence emission spectra for dimeric and monomeric forms after the pre-incubation with 15 mM of 2ME during 24 h. In this condition, all protein samples are monomers and the fluorescence emission spectra of the three aromatic residues (Fig. 6B) and their λ_max_ (Table 2) are similar. In order to quantify and compare the Trp and Tyr contribution to fluorescence emission, the spectra were normalized. As mentioned, the emission spectra of Tyr-Trp, Trp and Tyr in monomer (M) are twice time bigger than dimer (D) (Fig. 6C–E). However, the emission difference between monomer (M) and dimer (D) is biggest for Trp spectra (Fig. 6D) than for Tyr spectra (Fig. 6E).

**Table 2:** Fluorescence maximum emission wavelengths of StI W111C monomer and homodimer forms.

Fluorescence Emission Parameters In Solution				
λ_max_ (nm) Trp + Tyr residues				
Proteins	−AA−2ME	+AA−2ME	−AA+2ME	+AA+2ME
Monomer	334 ± 0.5	332 ± 1.0	334 ± 0.5	331 ± 0.6
Homodimer	334 ± 0.6	332 ± 1.5	334 ± 0.5	331 ± 0.7
λ_max_ (nm) Tyr residues				
Proteins	−AA−2ME	+AA−2ME	−AA+2ME	+AA+2ME
Monomer	319 ± 0.6	319 ± 1.0	322 ± 2.0	316 ± 1.0
Homodimer	322 ± 1.5	323 ± 1.0	316 ± 2.0	317 ± 2.0
λ_max_ (nm) Trp residues				
Proteins	−AA−2ME	+AA−2ME	−AA+2ME	+AA+2ME
Monomer	339 ± 0.6	337 ± 1.5	338 ± 0.6	333 ± 0.6
Homodimer	339 ± 0.5	338 ± 1.0	338 ± 0.5	334 ± 0.7

Fluorescence maximum emission wavelengths (λ_max_) for Trp and Tyr residues were calculated from spectra of monomer and homodimer oligomeric forms in solution (Fig. 6). Homodimer and monomer were analyzed by pre-incubating with 15 mM 2-mercaptoethanol (+2ME) during 24 h and without pre-incubation (−2ME). Fluorescence emission from oligomeric forms were also quenched with 300 mM acrylamide (+AA) and analyzed in the absence of quencher (−AA).

The conformational changes of StI W111C monomer and homodimer were analyzed using Trp emission as a function of acrylamide concentration. A considerable reduction of the fluorescence emission were observed (Fig. 6C–E and F,G) without displacement of λ_max_ for both molecular forms (Table 2). Similar quenching were observed for the reduced proteins (Fig. 6C–E, F,G and Table 2). The liner *Stern-Volmer* plots are typically of homogeneous population of the Trp residues (Fig. S7). The *Stern-Volmer* constant (K_SV_) for the monomer is 4.56 M^−1^ while for the dimer it is 33% lower (3.03 M^−1^) (insert Fig. S7).

### Tridimensional structure of StI W111C homodimers stabilized by disulfide bridge

A molecular model of StI W111C monomer was developed replacing Trp111 by Cys with MODELLER software using as template the RMN model n°1 of StI soluble structure. A disulfide-bridge rotamer library with 83 members was build from the PDBePISA web server (Table S in the Supporting Material). The variation of dihedral angles (*χ*^3^) around the Cβ-S-S-Cβ atoms for each disulfide-bridge rotamer was calculated. The results showed a wide conformational space of dihedral angles in correspondence with the prevalence of angles near to 90°, as has been described^57–61^ (Fig. S8A). According to these authors, most of disulfide-bridges has low strain energy (*χ*^3^ ≈ 90°) and are involved solely in structural stabilization. The 83 models of StI W111C homodimer were calculated using a rigid-body procedure. About 16 models were removed due to structural crashes. The remaining 67-homodimer models were aligned and clustered in nine structural groups by rmsd values with 8.0 Å as cutoff criteria (Fig. S9). Representative structures from clusters were selected considering the following three criteria: maximum molecular diameter between 5.5–6.5 nm in agreement with DLS studies, at least one Trp residue that participates in the protein-protein dimer interface formation (exposed previously in the monomer) with nearby quenching amino acid residues from other monomer in agreement with spectroscopy studies, good structural-quality.

Only eight models had a maximum molecular size between 5.5–6.5 nm (Fig. S8B) and all these models belong to cluster 4 (six models) and cluster 9 (two models) (Fig. S9). The model is identified by a number assigned during the modeling process and within parentheses are indicated the positions of each Cys residue, the chains and the pdb where the disulfide-bridge rotamer comes from. The model_63 (90a_90b_1grg), representative of cluster 4, and both models from cluster 9, model_14 (31a_32b_1bsr) and model_25 (32a_31b_11ba), accomplished the three criteria previously mentioned and were selected as starting models for molecular dynamics (MD) simulations in order to obtain more stable protein conformations.

**Figure 7:**
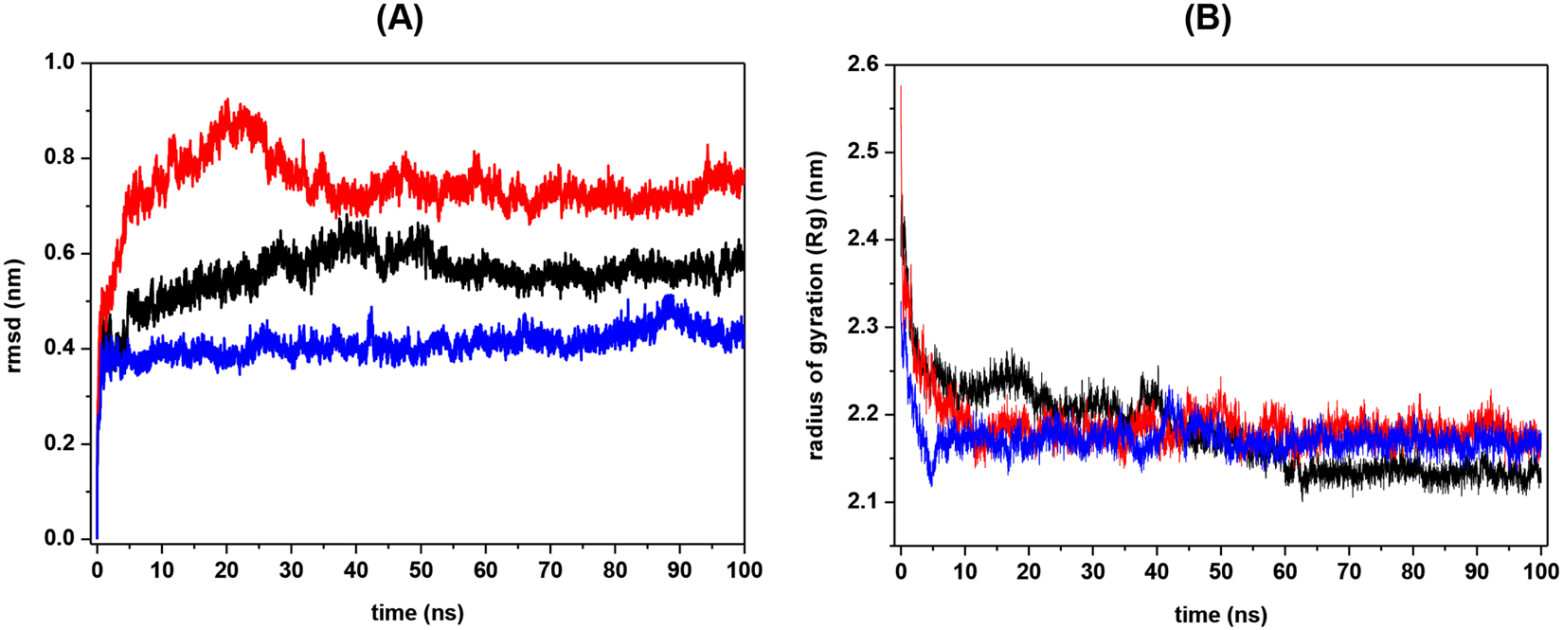
Stability and radius of gyration during molecular dynamics simulations of the three StI W111C homodimer structural models with the disulfide bond between Cys 111. Molecular dynamics simulations were carried using *GROMACS* simulation suite with *GROMOS96 43a1* force field. The root mean-square deviation (rmsd) of the backbone atoms **(A)** and Radius of gyration (Rg) **(B)** for the three StI W111C homodimer structural models are shown in nm: model_14 (black), model_25 (red) and model_63 (blue). The stability of all homodimer models was achieved after 40 ns of the trajectory.

The MD simulations from StI W111C homodimer model_14, model_25, and model_63 showed increase in the time-dependent backbone rmsd values relative to the starting structures at 300 K. The highest increase when reaching the equilibrium (rmsd plateau phase) was for model_63 (rmsd = 0.76 nm), followed by model_14 (rmsd = 0.58 nm), and the model_25 (rmsd = 0.44 nm) (Fig. 7A). On the other hand, the three simulations showed a decrease in the radius of gyration (Rg), with similar values for model_63 and model_25 (2.17 nm) and slightly smaller for the model_14 (2.13 nm) (Fig. 7B). For determine the structural/geometrical properties of protein-protein interfaces were performed analyses in PISA web server.

**Figure 8:**
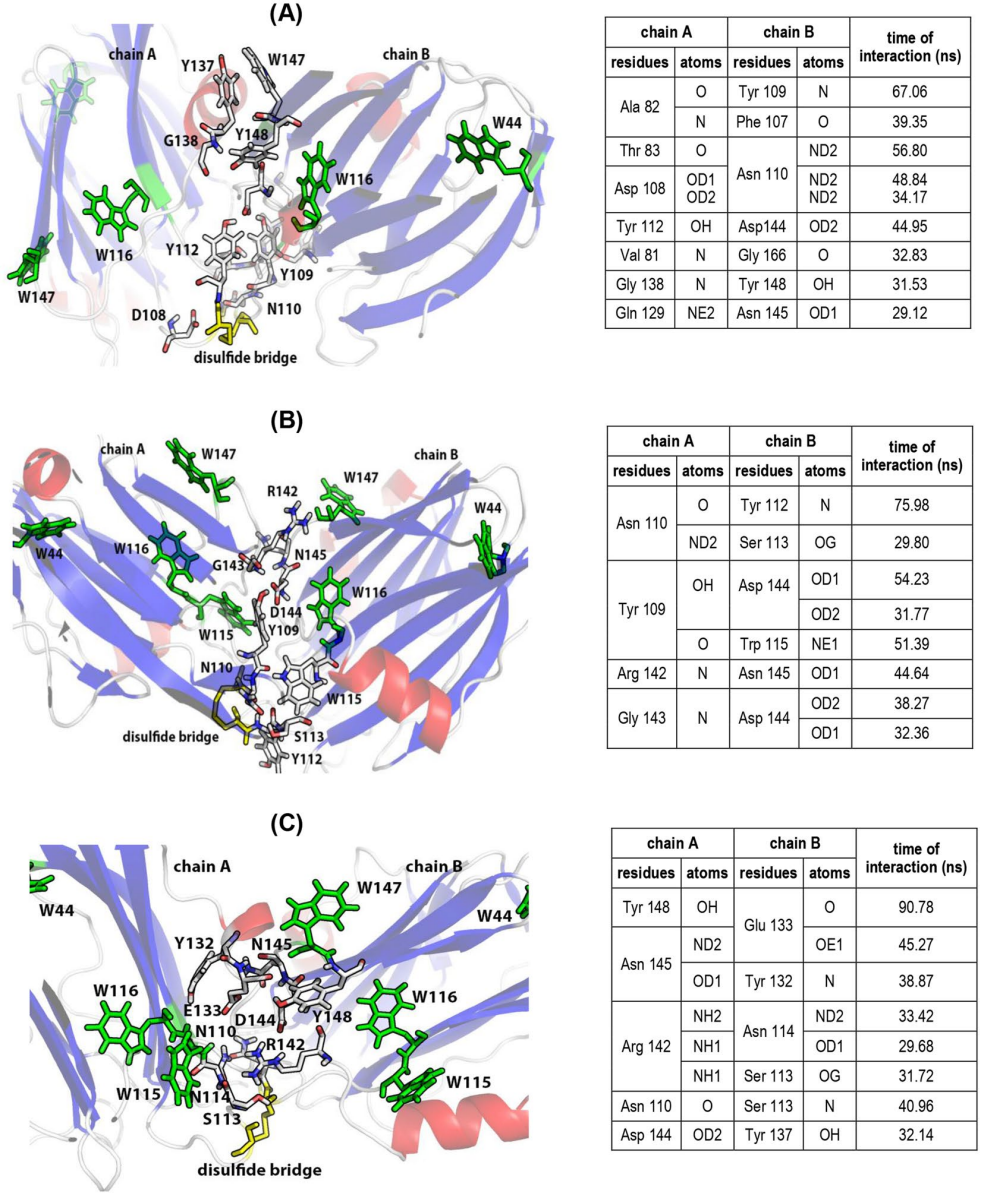
Inter-protomer interfaces of three StI W111C homodimer structural models stabilized by a disulfide bridge between Cys 111 during molecular dynamics. All homodimer models were achieved after 100 ns of the trajectory **(A)** model_14 (31a_32b_1bsr), **(B)** model_25 (32a_31b11ba), and **(C)** model_63 (90a_90b_1grg). The different elements of secondary structure are shown in red (helixes), blue (sheets) and gray (loops and unordered structures). Tryptophan residues (green), except W147 in model_14, and disulfide bridge (yellow) are also high-lighted. Tryptophan and amino acid residues involved in the protein-protein interaction are labeling. Tables inserted (right) summarize the hydrogen bounds established for more than 30 ns of the dynamics (>30% of the simulation time). Images were produced with the *Pymol v1*.*7*.*6*.*6* software for *Windows*^107^.

The model_14 (Fig. 8A) showed the highest increases after 100 ns of simulation in the following parameters: number of amino acids involved in the protein-protein interface (from 21 to 48), accessible area to interface (from 347.1 to 942.3 Å^2^), and gain solvation energy on complex formation (from −1.8 to −7.4 kcal/mol). The model_25 (Fig. 8B) showed increases in the number of amino acids involved (from 6 to 39), the interface area (from 84.6 to 700.2 Å^2^), and the gain solvation energy (from −4.0 to −8.4 kcal/mol). In the model_63 (Fig. 8C), increase in number of amino acids involved (from 23 to 40), the interface area (from 439.1 to 745.7 Å^2^), and the gain solvation energy (from −3.4 to −6.5 kcal/mol) were observed.

The Fig. 8 summarizes the amino acids that showed formation of hydrogen bonds during more than 30% of the simulation time for each models (Fig. 8 and inserted tables). In the model_14, there was an increase in the number of hydrogen bonds (from 3 to 16) and several amino acids with relevant functions in the pore-forming mechanism were identified in the protein-protein dimer interface. Several similar amino acids were identified in actinoporin lipid-binding sites determined and reviewed for different authors (R30, R52, S77, P80-G84, F107-Y112, S164)^11,14–17,20,62^. Other amino acids were identified in the RGD-site (R142, D144), in the protein-protein interaction site for pore oligomerization (N145, W147, T163, S164-A165) and in the carbohydrate-binding site (N129) described^17,19^ (Fig. 8A and inserted table). In model_25, the increase in the number of hydrogen bonds was from 2 to 9, and several amino acids were also identified in lipid-binding sites (R30, P80-V81, Y109-W115, S164, G166), RGD-site (R142-D144) and in pore oligomerization site (N145, W147, T163-G166) (Fig. 8B and inserted table). In model_63, the increase in the number of hydrogen bonds was from 6 to 8, and several amino acids were also indentified in lipid-binding sites (F15, R30, R52-R53, G79-V83, Y109-N114, S164, G166), RGD-site (R142-D144), in pore oligomerization site (N145, W147, T161-G169) and carbohydrate-binding site (Y132, E133, Y137) (Fig. 8C and inserted table).

## Discussion

The functional importance of several aromatic residues of Equinatoxin II (EqtII), an actinoporin from *Actinia equine*, for its insertion in membranes has been demonstrated^11,63^. These authors established that Trp112 and Tyr113 are the most important residues for SM recognition in lipid membranes by EqtII. Additionally, mutants of EqtII for these positions (W112 to L, E, R, F or A and Y113 to F or A) corroborated the relevance of the aromatic residues for pore formation mechanism^11^. In sticholysins, the equivalent positions (StII Y111N and StI W111C) were also mutated with decreases in pore-forming activity^38,64^. Despite of Trp112 functional importance in EqtII, it is not a conserved residue in the actinoporin family. The inspection of twenty-one known actinoporin amino acid sequences with hemolytic activity shows that Trp112 of EqtII is substituted for Leu (57%) or Phe (9%)^20^.

A single Cys residue in the monomeric actinoporins could be sufficient to induce spontaneously self-dimerization by a disulfide bridge formation. This effect has been reported for different single Cys actinoporin mutants: EqtII K77C, EqtII R126C, EqtII A179C^41^, StI E2C, StI F15C, StI R52C^39^, StI W111C^38^ and StI P80C^42^. The tendency to self-dimerization by a disulfide bridge of StI E2C, StI F15C, StI R52C and StI W111C mutants are correlated with solvent exposure of each mutated positions in the protein structure^39^. Specifically, Trp111 is the most exposed in StI structure; therefore this StI W111C mutant shows the highest tendency of spontaneous self-dimerization by disulfide bridge: 90% of StI W111C formed dimers spontaneously after 24 h of incubation at room temperature^38^. The c(s) curve for StI W111 C (Fig. 1C, left) corroborate the large homogeneity and abundance of the homodimer fraction, and that StI W111C presents high spontaneous self-dimerization tendency by disulfide bridge formation in solution. The major intensity of second peak (92%) is in agreement with the previous studies^38^.

Additionally, the SV-AUC results show that StI W111C only exists in monomeric and dimeric forms, independent of each other in solution, at least below 24.4 µM protein concentrations. However, large particles such as aggregates larger than dimers present in eluted fractions cannot be detected by analytical ultracentrifugation due to the quickly sedimentation at the high rotor speeds. Therefore, DLS were used to show that large aggregates (Fig. S5A) were present in insignificant quantities according to the mass distributions (Fig. S5B). The main contributions for scattering in the StI W111C dimer and rStI (Table in Fig. S5) came from mono-disperse distribution (Pd = <20%, Table 1), indicating a homogeneous sample. The apparent MW of StI W111C in monomeric and dimeric forms estimated from DLS experiments closely matches those determined by theoretical approach from the Expasy server (Table 1), MALDI-TOF-MS measurements (Fig. S3 and Table 1), SV-AUC experiments (Fig. 1 and Table 1) and SDS-PAGE (lanes 2 in Figs 2B and 3B). Therefore, DLS and SV-AUC results indicate that the homodimer is not self-aggregated or aggregated with the monomer. The difference of stokes radius by SV-AUC and the hydrodynamic diameters by DLS for the dimer, monomer and rStI are evident (Table 1). However, the increase in StI W111C DLS hydrodynamic diameter between monomer and dimer is 1.7 nm or 40% and the same increase in the SV-AUC stokes radius is 1.8 nm or 50%. This shows that although the difference in individual values for molecular diameter or radius can be large depending on the method, the variation in the increase from monomer to dimer is proportional independentment of the method used.

Previously, the StI W111C dimer stabilized by disulfide bridge and an irreversible dimeric variant, cross-linked with homo-bifuncional bis(maleimide)-hexane reagent, have only been partially purified by SEC chromatography^38,46^. The small MW estimations by SEC chromatography using *Superdex 75* (Fig. 3 and Table 1) are due to protein interactions with the resins, as demonstrated by previous studies where a marked retardation in the actinoporin elution volume were associated with non-specific^52,65,66^ and specific^19^ interactions with the resins. In the later case, it was demonstrated that FraC actinoporin binds reversibly to *Superdex 200* resin, which is composed of carbohydrates dextran and agarose^19^. More specifically, the reported FraC/N-acetyl glucosamine crystallographic structure complex showed a sugar binding site that overlaps with one of the lipid binding sites. Furthermore, a double mutant of FraC (W112R/W116F), which includes the equivalent position of Trp111 in StI, decreases the interaction with the *Superdex* resin^19^. In agreement with these results, the comparison of the rStI and StI W111C SEC results indicate that the substitution of Trp111 by Cys in the monomers decreases the interaction with the matrix but does not eliminate it, suggesting the involvement of Trp111 residue of StI in the carbohydrate binding site. The dimerization by disulfide bridge does not eliminate the interaction with the matrix, thus, limited access to the sugar binding sites is still available. Considering the results, it is not recommended to use the SEC chromatography for MW estimates actinoporins.

Another problem with SEC chromatography of actinoporin Cys-mutants is the difficulty in fully separating monomer-dimer mixtures. Therefore, an ion-exchange SP-chromatography was optimized for StI W111C dimer purification (Fig. 2A). A well resolved dimer eluted in peak II between peaks I (monomer), III (mostly monomer), and IV (monomer). Further confirmation of the oligomeric identities in each peak was obtained with SEC *Superdex 75* and electrophoretic migration (Fig. 3). The monomeric species present in peaks I and IV showed a peculiar behavior since they were quite similar in their SEC elution profile (Fig. 3A) and electrophoretic migration (Fig. 3B), but had different profiles in cation-exchange (Fig. 2A). This result suggests that these monomeric species have different electrostatic distribution in their structures, probably due to conformational or physicochemical modifications produced during the extraction and purification processes.

The HA of StI W111C monomer and dimer forms were estimated from the time course of erythrocyte lysis by measuring the decrease in the turbidity (DO_600 nm_) of an erythrocyte suspension (Fig. 4A,B). The results indicate that StI W111C dimer is 193 times less active than monomer according to HC_50_ values, and suggest that dimer has a hemolysis mechanism less efficient and/or takes a longer time for pore-assembling when compared to monomer. Reduced HA of a StI W111C dimer solution, with low amounts of contaminating monomer, with respect to rStI protein, has been previously reported^38^. According to these authors, the reduced HA of the StI W111C is related to the incapability to associate with membranes due to the Trp111 mutation to Cys in the membrane-binding site and as a consequence of a disulfide bridge formation near the membrane-binding site. Surprisingly, StI W111C homodimer is active (interval of 30–400 nM) according to our previous findings, but in contrast with the results published by other authors using protein concentrations lower than 70 nM^38^.

Purification of StI W111C dimer with high purity guaranteed their accurate conformational characterization. The results suggest that replacement of Trp111 by Cys and the disulfide bridge presence do not lead to changes in the secondary-structure content of StI W111C monomer and dimer compared to rStI (Fig. S6A, inset). The unfolding process of StI W111C monomer and dimer does not occur in a single step with the presence of only native or denatured conformations, instead a significant accumulation of spectrally distinct conformational intermediates are detected (Fig. 5A,B). As verified by Far-UV CD (Fig. 5C) and fluorescence emission (Fig. 5F) spectroscopy, rStI presented conformational changes but the complete pattern of the unfolding process is not observed. The spectroscopy results suggest that the thermal stability of StI W111C monomer and rStI according to Tm (Fig. 5I) are identical and similar to those reported for EqtII and StII^37,67,68^, whereas StI W111C dimer is less stable. From analysis of the Tm values (Fig. 5I), it is noteworthy that a thermal structural transition concerning secondary structural changes occurs before the conformational transition in tertiary structures. This results suggest a native-to-partially unfolded transition with a concomitant loss of native secondary structure and its rigid tertiary structure with a partially exposition of tryptophan residues.

Since StI W111C monomer has four Trp and thirteen Tyr residues located at different positions, their fluorescence spectroscopic characterization could provide information about dimer conformation. The fluorescence spectroscopy characterization indicates that the dimerization by disulfide bridge is responsible for the lower fluorescence emission of the dimer and that the 2ME has a quenching effect on fluorescence emission (Fig. 6A,B). When disulfide bridge was reduced with 2ME, the Trp (Fig. 6D) and Tyr (Fig. 6E) integrated spectra were similar for monomer and dimer. A lower emission of the homodimer is a consequence of disulfide bridge or side-chains nearby the Trp on the homodimeric protein-protein interface, with fluorescence quenching properties. The protein fluorescence spectral characteristics reflect the Tyr and Trp average exposure to solution, and fluorescence emission additionally could be subject to quenching by iodide, acrylamide, and nearby disulfide groups^69^. The fluorescence quenching of Trp by acrylamide, show a lowest surface-exposure average degree for Trp in the dimeric form (Fig. 6) indicating that Trp participates in the protein-protein interface formation.

Disulfide bridges are known to contribute to the Near-UV CD spectra with broad weak signals throughout the spectrum^53,70–72^ and their locations are critically dependent on the dihedral angles around the Cβ-S-S-Cβ atoms (*χ*^3^)^72–79^. Depending upon *χ*^3^ values, the disulfide bridges have two chiralities with inherent optical activities: P (0° < *χ*^3^ < 180°, turn right-handed sense) and M (0° > *χ*^3^ > −180°, turn left-handed sense). For a disulfide bridge, two n-σ* transitions are possible according to the symmetries *a* and *b* of the lone-pair molecular orbital: na-σ* and nb-σ*. For cis (*χ*^3^ = 0°) and trans (*χ*^3^ = ±180°) conformers, according to the symmetry selection rule, the chromophores are no longer inherently dissymmetric and their optical activity can derive only from their dissymmetric environment^77^. Since the CD is dependent on the transition polarization, in the case of P chirality the nb-σ* transition leads to a negative band, while the na-σ* transition leads to a positive band, and viceversa for M chirality^77^.

Unfortunately these na-σ* and nb-σ* transitions cannot be experimentally discriminated and the chirality attributable to disulfide bridge conformers from CD data alone is therefore ambiguous without a knowledge of the *χ*^3^ angle. However, a qualitative analysis of CD band signal can be made using an empirical “*quadrant rule*”^77^ to predict the probably *χ*^3^ angle value and disulfide bridge chirality (P or M). The spectral difference between StI W111C dimer and monomer is shown in Fig. S6B (dash line) and it observed that the disulfide bridge could give rise to a broad positive band around of 270–300 nm region with a maximum wavelength at 275–280 nm. According to “*quadrant rule*” this positive band could be due to the absorption of a 85° (chirality P) or −95° disulfide bridge with *χ*^3^ angle value of (chirality M)^77^. The disulfide chirality can be determined by molecular modeling, NMR or crystallography techniques. The three initial models used for MD simulations achieved conformational stability and showed a moderate structural deviation or global fluctuation respect to starting structure (Fig. 7A). Additionally, the homodimer dimensions decrease (Fig. 7B) for a structural packing by increasing the number of interatomic interactions during the MD simulations. The illustrative representation of the relative protomers orientation in the dimer models interfaces are show in Fig. S10. In the model_14 and model_63, alterations in the membrane-binding site structure (decrease in the length of the helix α2 in one monomer) were observed and at least one POC-binding site (POC-1) and carbohydrate-binding site (NGY-1) are involved in homodimer formation (Fig. S10A,C). These models could explain why the ability to form pores of StI W111C homodimer (Fig. 4A) and their interaction with the *Superdex* carbohydrate-matrix (Fig. 3A) are decreased. Contrary, in the model_25 the POC and NGY binding sites do not participate in the protein-protein interface (Fig. S10B), therefore, this model could fully interact with membranes and *Superdex* column. In addition, the MD simulation of the three homodimer models indicated the participation of acid residues (D108 and D144) in the interface of model_14 (Fig. 8A inserted table), which interact by hydrogen bonds with Asn and Tyr residues, respectively. In contrast, in the two remaining models both basic and acidic residues are involved (Fig. 8B,C inserted table). This interaction in model_14 might explain the strengthening of the positive distributions of charges in the dimer that justify its subsequent elution after monomer in *Sepharose-SP* (Fig. 2A). Only model_14 accomplished the second criterion related to the Trp residue exposure in the monomer that participates in the protein-protein interface formation with nearby quenching amino acid residues from other monomer according to spectroscopy studies. Specifically, the W147 of a monomer remains during the last 60% of dynamic time in the vicinity of the Y137 of another monomer probably establishing π-π interactions (Fig. 8A). This interaction could be the cause of the decreased Trp fluorescence emission observed for dimer in the spectroscopy results (Fig. 6). As previously reported, the amino acid side chains of Cys, His, Tyr and Phe are able to quench Trp fluorescence and phosphorescence^80^. This fluorescence quenching could occur by electron transfer from the ^1^L_a_ state of Trp to the Tyr residue equivalently to internal conversion between two excited states. Analysis of the disulfide-bridge dihedral angles during the three MD simulations showed that the *χ*^3^ average value (87°) (Fig. S8C) for model_14 is similar to one of the two values (85° or −95°) estimated by the “*quadrant rule*” from the Near-UV CD data (Fig. S6B). There is possible to make a P-chirality (right-handed helix) assignment for the model_14 from the positive signal of the Near-UV CD band and *χ*^3^ value. Meanwhile, for model_25 and model_63 the *χ*^3^ average value (102°) (Fig. S8C) were higher than the estimated, compatible with an observed negative signal of the Near-UV CD band at 270–280 nm. Therefore, the model_14 is in agreement with the spectroscopic data in the conformational dimer characterization.

The findings will contribute to the advancement of the design and development of the molecular delivery systems to cellular cytosol based on pore-forming proteins from sea anemones. The procedure for effective dimer purification described here is the first step to fully characterize their structure and mode of action. In order to provide new insights into the structure and pore-forming activity of the dimer we demonstrated that purified dimer is 193 times less hemolytic than the monomer. Our spectroscopy results showed that Trp/Tyr residues participate in homodimerization and that the dimer is less thermostable than the monomer. The constructed three-dimensional model of the dimer indicates that Trp147/Tyr137 are the residues at the homodimer interface. Spectroscopy results validated the 3D-model and estimated the disulfide-bridge dihedral angle responsible for dimerization. The homodimer model suggests that alterations in the membrane/carbohydrate-binding sites in one of the monomers could explain the decrease in the homodimer ability to form pores.

## Methods

### Expression and purification of StI W111C mutant

The stIW111C gene expression was performed by auto induction method^81^ using BL21 (DE3) *E*. *coli* strains transformed by heat shock method^82^ with the pUC19-stIW111C plasmid^38^. Flasks containing 600 mL of auto induction media (ZYB-5052) were inoculated with Non-inducing (MDG) culture and grown overnight at 37 °C and 250 rpm. Appropriate antibiotic selection was performed for each media. Protein expression was analyzed by sodium dodecyl sulfate polyacrylamide gel electrophoresis (SDS-PAGE) using molecular weigth marker^83^. StI W111C was purified from supernatants of lysed bacteria using ion-exchange chromatography on a carboxymethyl cellulose (CM-52 Whatman, EUA) column as previously described^39^.

### Purification of StI W111C homodimers

Purification of StI W111C homodimers was carried out from carboxymethyl-cellulose eluted fractions using *Sepharose-SP Fast Flow* ion exchanger (*Amersham Biosciences*, Sweden). The protein elution was performed with a linear gradient of 10–60% of 0.5 M NaCl in PBS (0.02 M Na phosphate pH 7.4). Monomers and dimer of StI W111C were identified by SDS-PAGE^83^ and purity analyzed by high performance liquid chromatography on a reverse phase (HPLC-RP) (*Shimadzu*, Japan) with *RP-C4 UltraC4* column (*RESTEK*, USA) as previously described^39^. Absorbance at 280 nm (A_280 nm_) and fluorescence emission at 334 nm, after excitation at 295 nm, were used for monitoring the chromatography procedure.

### Molar extinction coefficient and protein concentration determinations

The molar extinction coefficient (ε) of StI W111C homodimers stabilized by disulfide bridge were determined by linear fit of plotting the absorbance measurements increase at 280 nm (A_280 nm_) as a function of protein concentration. An *Ultrospec 4000 UV/Visible* Spectrophotometer (*Pharmacia Biotech*, Suecia) was used for two different procedures: (i) point measuring, and (ii) corrected absorbance considering the scattering contributions at 350 nm (A_350 nm_) (Eq. 1).

1 $${{\rm{Acorr}}}_{280{\rm{nm}}}={{\rm{A}}}_{280{\rm{nm}}}-{{\rm{A}}}_{350{\rm{nm}}}$$

Protein concentration was determined by the method of Lowry^47^ using a standard solution of crystalline bovine serum albumin (BSA) (Sigma, *Steinheim*, Germany). BSA (range 0.1–1 mg/mL) and measuring A_620 nm_ after 30 min in a *Multiskan EX microplate* reader (*Labsystems*, Finlandia).

### Molecular weight and size determinations

Mass spectra were measured by MALDI-TOF on an Autoflex Speed TOF/TOF (*Bruker Daltronics GmbH* Bremen, Germany) using nitrogen laser at 337 nm operated in linear positive mode with acceleration of 19 kV. Analyses were performed from 1 µL of purified protein mixed with 3 µL of a solution of α-Cyano-4-hydroxycinnamic acid (HCCA) prepared at 10 mg/mL in TFA 0.1%/ acetonitrile 30%. The mixture was added directly onto MALDI target plate *MTP Ground Steel*. The equipment manipulation and result analysis were performed by the *Flex Control software v3*.*4*, and the theoretical masses were calculated using the Isotope *Pattern software v3*.*4* (Build 76, *Bruker Daltronics GmbH* Bremen, Germany). The sedimentation velocity experiments using analytical ultracentrifugation (SV-AUC) were performed at 42 000 rpm in a *Proteome-Lab XL-A/XL-I* analytical ultracentrifuge (*Beckman Coulter*, USA) equipped with a 4-hole titanium *An-60 Ti* rotor and cells with a double channel centerpiece (*Beckman*, USA) at 20 °C in the vacuum. The A_280 nm_ scan data was acquired with 20 µm radial resolution, at 5 min intervals for each sample reading. Protein solutions of 24 and 12 µM were used in TBS buffer. The *SEDENTERP* software (http://www.rasmb.bbri.org) was used to estimate the partial specific volume of the protein (*υ*), the density (*ρ*), and the viscosity (*η*) of the TBS buffer. The collected radial scans were analyzed using the sedimentation coefficient [c(s)] continuous distribution analysis model by the *SEDFIT v14*.*7* software^84^. The proteins’ MW and c(s) distribution were calculated using the global fitting with species analysis model of the *SEDPHAT* software^85–87^. All parameters were allowed to float freely and then submitted to statistical analyses by *Monte-Carlo* non-linear regression with, at least, 200 iterations and a confidence level of 0.68. The molecular species percentage was obtained by integrating the range of sedimentation coefficients, from peaks identified by the *SEDFIT* software. The c(s) was calculated using the appropriated correction for water viscosity and density at 20 °C (S_20,w_). Each peak eluted from SP-chromatography were analyzed by size-exclusion chromatography (SEC) on a *Superdex 75 10/300 GL* column (*GE*, USA) using a flow rate of 0.8 mL/min. The procedure was performed through isocratic chromatography using TBS buffer on an *AKTA purifier* chromatography system (*GE*, USA). Chromatography was monitored at A_280 nm_ and protein was verified by 15% SDS-PAGE^83^.

DLS measurements were made on a *Zetasizer Nano Series Nano-ZS* (*Malvern Instruments*, UK) for particle size determination at 20 °C. He-Ne laser with λ = 633 nm was used for measurements at 173° backscatter detection, using *PCS8501* glass cuvette. Additional parameters used for measurements were: material = protein (RI = 1.45, absorption = 0.001), dispersant = PBS with 150 mM NaCl (viscosity 0.9223 cp, RI = 1.332).

### Hemolytic activity assays

The hemolytic assay protocol was approved prior to experiments by the ethics committee of the Institute of Foods and Pharmacy, University of Havana, which is the Institutional Review Board of this University. Human red blood cells (HRBC) were collected intravenously in citrate in conformity with the recommendation provided in the Code of Ethics of the World Medical Association (Declaration of Helsinki) for experiments involving humans in the Institute of Foods and Pharmacy, University of Havana (Cuba). The subjects provided verbal consent following the explanation of the blood sampling procedure, which involved only minimal risk. Hemolytic activity (HA) was determined by measuring the turbidity at 650 nm with a microplate reader *Multiskan EX* (*Labsystems*, Finlandia) as previously described^39^. The time course of hemolysis was followed for 15 min at room temperature and the percentage of hemolysis was determined by Eq. 2:

2 $${\rm{Hemolysis}}\,( \% )=({{\rm{A}}}_{0}-{{\rm{A}}}_{15{\rm{\min }}})/{{\rm{A}}}_{0}-{{\rm{A}}}_{{\rm{TX}}100})\,\times \,100$$

where A_0_ and A_15 min_ represent the A_650 nm_ for 0 and 15 min assays, respectively. A_TX100_ is the value of completely lysed HRBC by adding Triton X-100 (*Sigma*, EUA) at 1 mM final concentration. In order to characterize the HA, the percentages of hemolysis of each protein as a function of the amount of toxin added were considered; the data was when adjusted to the *Hill* function. The *k* parameter of *Hill* sigmoidal function was estimated as the protein concentration necessary to achieve 50% hemolytic activity (HC_50_).

### Structural characterization

Far-UV (190–260 nm) and near-UV CD (250–350 nm) spectra were recorded on a *JascoJ-815* spectropolarimeter (*Jasco Corporation*, Tokyo, Japan) equipped with a *Peltier* thermostatic controller. Spectra were obtained in 1 and 10 mm path-length as previously described^39^. The ellipticity readings for each wavelength (θ_λ_) were converted into mean residue ellipticity [θ]_λ_ according to Eq. 3:

3 $${[{\rm{\theta }}]}_{{\rm{\lambda }}}=100\,{{\rm{\theta }}}_{{\rm{\lambda }}}/{\rm{c}}\,{\rm{n}}\,{\rm{L}}$$

where: θ_λ_ is the ellipticity (degrees°); n, the number of amino acid residues; c, the protein concentration (mM) and L, the path-length cells (cm). The value of [θ]_λ_ is expressed in degrees.cm^2^/dmol^73^. The secondary structure contents of the proteins were estimated by *CONTIN* program^88,89^ using the reference protein set-SP175 in the *Dichroweb* Internet server^90–92^.

Fluorescence spectra of proteins were measured in a *Shimadzu RF-5301PC* spectrofluorophotometer (*Shimadzu Corporation*, Kyoto, Japan) using 1 cm pathlength quartz cuvettes^39^. Excitation and emission slit widths of 5 nm were used. Emission spectra of proteins (1 µm) were recorded from 300 to 450 nm after excitation of tryptophan at 295 nm. Selective quenching of the Trp fluorescence emission was achieved by adding increasing acrylamide concentrations^39^. Intrinsic fluorescence measurements of proteins were also performed with (15 mM) and without 2ME as previously described^39^.

Thermal stability of proteins (20–90 °C) was determined by measuring simultaneously the protein secondary structure by Far-UV CD (220 nm) and tryptophan fluorescence emission at 334 nm after Trp-excited at 295 nm. A *Jasco 1500* spectropolarimeter (*Jasco Corporation*, Tokyo, Japan) was used with transparent four-side quartz-covered cuvettes of 10 mm pathlength at a 100 nm/ min scanning speed and constant stirring at 200 rpm. Heating rates were performed in steps of 10 °C, with 1 min equilibration time. The transition curves were normalized to the fraction of the folded/unfolded protein using thermal denaturation multi analysis program (*Jasco Spectra Manager*, Jasco Corporation, Japan) by the standard equation (Eqs 4 and 5, for CD and fluorescence, respectively):

4 $$ \% \mathrm{CD} \mbox{-} \mathrm{denaturation}=(({{\rm{\theta }}}_{20}-{{\rm{\theta }}}_{{\rm{temp}}})/({{\rm{\theta }}}_{20}-{{\rm{\theta }}}_{90}))\,\ast \,100$$

5 $$ \% F \mbox{-} \mathrm{denaturation}=(({{\rm{F}}}_{20}-{{\rm{F}}}_{{\rm{temp}}})/({{\rm{F}}}_{20}-{{\rm{F}}}_{90}))\,\ast \,100$$

where: θ_20_ (F_20_) and θ_90_ (F_90_) represent the ellipticity or fluorescence values for fully-folded and fully-unfolded species, respectively; and θ_temp_ (F_temp_) is the observed ellipticity at 220 nm or fluorescence at 334 nm^93,94^. Melting temperature (Tm) values corresponding to the temperature at the midpoint of the monophasic thermal transition were calculated as the maxima of the first derivatives of the percentage of change as a function of temperature.

### Modeling of the three-dimensional (3D) structure of StI W111C homodimers stabilized by disulphite bridge

A 3D model of StI W111C mutant was constructed using the *MODELLER software v9*.*10*^95^. The Glu16 and Trp111 residues from the NMR experimental StI structure (Protein Data Bank-PDB code 2KS4, model 1)^96^ were substituted by Gln and Cys, respectively, according to the StI W111C sequence. The model was validated using *Ramachandran* plot from *PROCHECK* server (http://services.mbi.ucla.edu/PROCHECK/)^97^. To construct a 3D model of the StI W111C homodimer covalently bound by a disulfide bridge, the experimental conformations of interchain disulphide groups within homodimeric interfaces was first investigated. The PDB database (http://www.pdb.org) was filtered using the *PISA web server* (http://pqs.ebi.ac.uk/) to yield all homodimer structures containing at least one disulfide bridge in their interfaces. A disulfide bridge library was built by extracting the tridimensional coordinates of all pairs of oxidized cysteine residues present in the homodimer interfaces of several high-resolution structures. Each one of these pair-wise coordinates was used to align the mutated Cys111 residue from two StI W111C monomer models by a rigid-body global search, using the *Chimera software v1*.*10*.*1*^98^. After the alignments, the coordinates from the mutated Cys111 residues (from each monomer) were substituted by the coordinates of the oxidized cysteine residues, as to generate an homodimer model of StI W111C for every disulfide bridge in the library. The homodimer models were minimized and their structural qualities were analyzed by the *WHAT IF* web server (http://swift.cmbi.ru.nl/servers/html/index.html); models presenting atom clashes were discarded. The remaining models were then clustered using a hierarchal clustering algorithm with a root mean square deviation (rmsd) cutoff of 8.0 Å by *MaxCluster software*^99^. Clusters were evaluated by comparing their structural features to the experimental results obtained from DLS, CD and fluorescence spectroscopy measurements. Several representative structural models were selected according to structural-quality and spectroscopies criteria and employed as starting models for molecular dynamics (MD) simulations.

The structures were solvated in a periodic cubic box filled with about 33000 single point charge water molecules^100^. The minimum distance between any protein atom and box edges was set to 1 nm. In order to neutralize the overall charge of the system, six chloride ions were added. Subsequently, all the systems were subjected to a steepest descent energy minimization algorithm until a tolerance of 100 kJ/mol was reached. MD simulations were performed using *GROMACS software package v5*.*1*^101–103^ with the *GROMOS96 43a1* force field implemented on a parallel architecture^104^ (amino acids ionization was automatically adjusted to a neutral pH environment (pH = 7.0). Each MD system was subjected to a 100 ns simulation at 300 K using the isochoric isothermal (NVT) ensemble. The *LINCS* algorithm^105^ was used to constrain bond lengths and the integration time step was 2 fs. The long-range electrostatic interactions were treated until 1.2 nm using the particle-mesh *Ewald* method (PME)^106^ with short-range cutoff of 0.9 nm. A twin-range cut-off was used for the calculation of *van der Waals* (VDW) interactions, with short-range and long-range cut-off radius of 1.0 and 1.4 ns, respectively; the non-bonded pair list was updated every 10 steps. *Gmx-rmsd* and *gmx-gyrate* were used to obtain the backbone rmsd and radius of gyration (Rg), respectively. The protein internal hydrogen bonds were detected using the *GROMACS tool gmx-hbond* tool and their time of interaction during trajectory was calculed using a perl script by Justin Lemkul (http://www.bevanlab.biochem.vt.edu/Pages/Personal/justin/scripts.html). The visual analyses of MD trajectories and protein structures were carried out using *PyMol* software^107^ (http://www.pymol.org). The geometrical characterization of StI W111C homodimer protein-protein interfaces was carryout with *PDBePISA web server* in Protein Data Bank in Europe (http://www.ebi.ac.uk/pdbe/pisa/).

## Electronic supplementary material

Supplementary information
