# Supplementary material for: Self-homodimerization of an actinoporin by disulfide bridging reveals implications for their structure and pore formation
Source: Sci Rep. 2018 Apr 26;8:6614. doi: 10.1038/s41598-018-24688-2 (PMC5920107; doi:10.1038/s41598-018-24688-2)
Supplement: Supplementary file 1 — Supplementary information [file 41598_2018_24688_MOESM1_ESM.pdf]

Self-homodimerization of an actinoporin by disulfide bridging reveals implications for their structure and pore formation

Aisel Valle Garay*, Luis Benito Pérez Socas, Liem Canet Santos, Yadira de la Patria Hervis Valdés, German de Armas Guitart, Diogo Martins-de-Sa, Jônatas Cunha Barbosa Lima, Adolfo Carlos Barros Souza, João Alexandre Ribeiro Gonçalves Barbosa, Sonia Maria de Freitas, Isabel Fabiola Pazos Santos.

* Corresponding author.

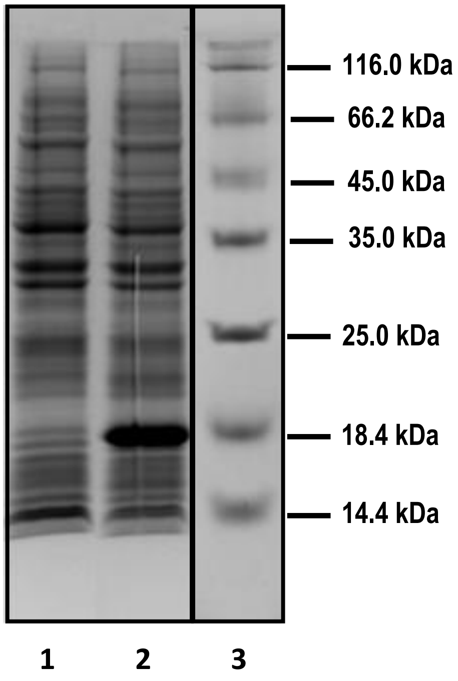

Supplementary Fig. 1: Assessment of StI W111C mutant expression.

The *pUC19-stIW111C* expression vector was used to transform *BL21(DE3) E.coli* cells. StI W111C gene expression was induced by auto-induction method as previously described [81] and analyzed by 12%SDS-PAGE according to [83]. Lane 1: total proteins from non-induced *E. coli* cells (MDG media), lane 2: total proteins from induced *E. coli* cells (ZYB-5052 in auto-induction media), lane 3: Pierce unstained protein molecular weight marker (*Thermo Scientific*, USA). The figure shows lanes that were non-adjacent in the gel and they are being indicated by a black line delineating their boundary.

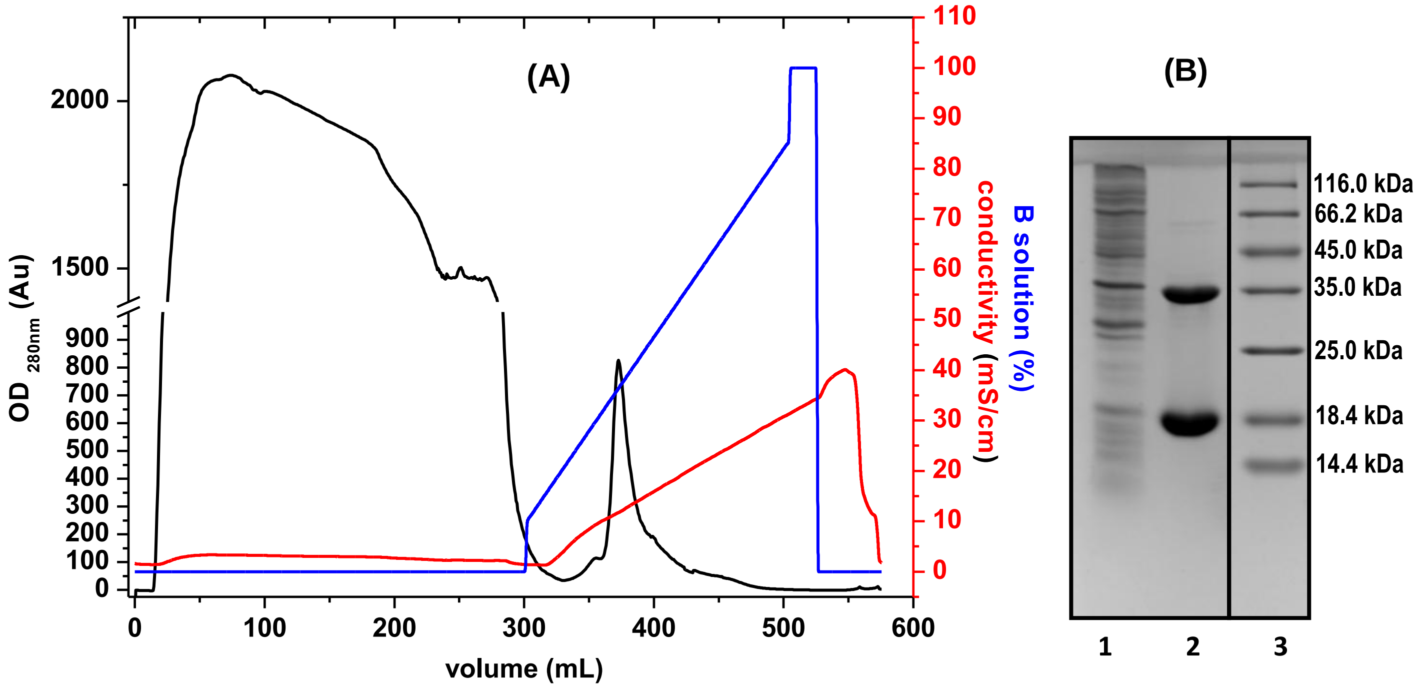

Supplementary Fig. 2: Purification of StI W111C from supernatant of bacterial lysed.

(A) Profile of ion-exchange chromatography of the StI W111C mutant on a carboxymethyl cellulose (CM-52) column (r/L: 0.8/20 cm). Purification was carried out from supernatant of lysed bacteria, as previously described [39]. The procedure was performed at 20.9 cm/h lineal flow and 6 mL fractions on *ÄKTAprimer plus* chromatography system (*General Electric*, Sweden). Absorbance at 280 nm (OD_{280nm}) (black line), gradient of NaCl as percentage of solution B during elution (blue line) and conductivity (red line). **(B)** SDS-PAGE 12% of acrylamides according to [83] without heat and reducing agent. Lane 1: unbound protein fractions; lane 2: StI W111C eluted fractions, and lane 3: Pierce unstained protein molecular weight marker (*Thermo Scientific*, USA). The figure B shows lanes that were non-adjacent in the gel and they are being indicated by a black line delineating their boundary.

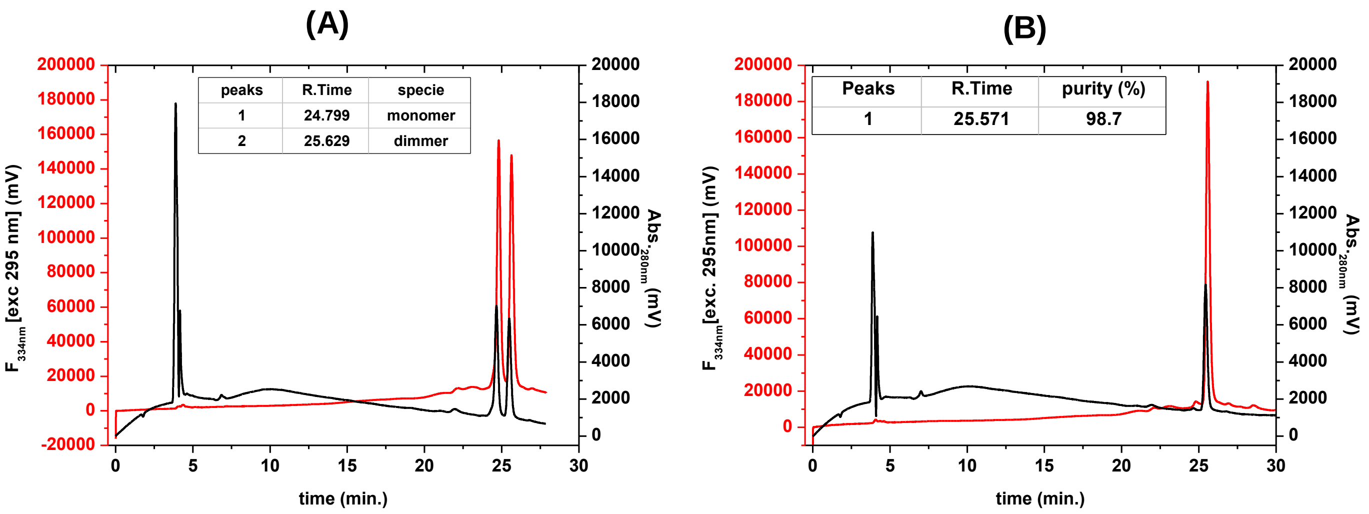

Supplementary Fig. 3: Protein purity analysis by HPLC.

(A) Profile of HPLC-RP of the StI W111C mutant from eluted fractions in CM-52 cation-exchange chromatography (Supplementary Fig. 2). Two protein peaks corresponding to monomer and dimmer at 24.8 and 25.6 min of retention time (insert table), respectively were observed. **(B)** Purity analysis of StIW111C homodimers stabilized by disulfide bridge from eluted fractions in *Sepharose-SP* cation-exchange chromatography (Fig. 2). A single protein peak at 25.6 min corresponding to dimmer was observed (insert table). Chromatography were carried out on a *RP-C4 UltraC4* column (5 µm, r/L: 4.6/150 mm) (*RESTEK*, USA) with a *HPLC prominence UFLC Shimadzu* system consisted of a *CBM-20A* comunications modules, *DGU-20A5* degasser, *LC-20AD* liquid chromatography pump, *SPD-M20A* diode array prominence for uv/visible and fluorescence detect, *CTD-20A* column oven and LC solutions program at 37˚C and 1 mL/ min of flow. The protein solution (0.75 µg/mL) was injected to column and washed with 5 mL of 0.1% trifluoroacetic acid (TFA). Protein elution was carried out with a linear gradient (30 min.) since 0-100% acetonitrile in 0.1% trifluoroacetic acid (TFA). Absorbance at 280 nm (Abs_{280nm}) (black line) and fluorescence emission at 334 nm (red line) after excitation 295 nm were used for monitored the chromatography procedure.

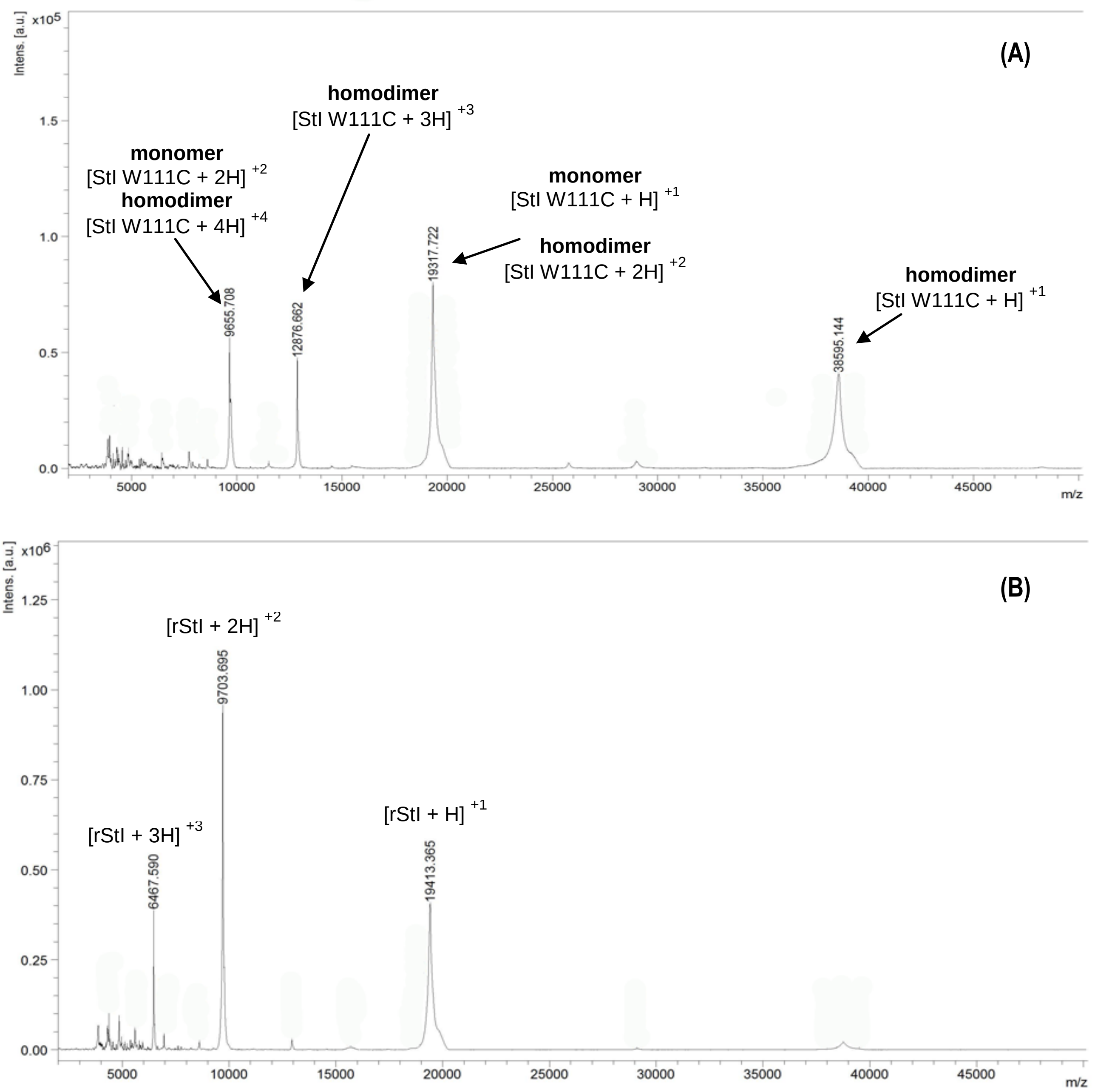

Supplementary Fig. 4: MALDI-TOF MS spectra profile of StI W111C and rStI from 2000 to 50000 m/z.

MS spectra for StI W111C **(A)** and rStI **(B)** proteins purified by carboxymethyl cellulose (CM-52) ion-exchange chromatography. The possible m/z distributions for both different oligomeric and charge states are identified. The intensities are expressed in arbitrary units (Au).

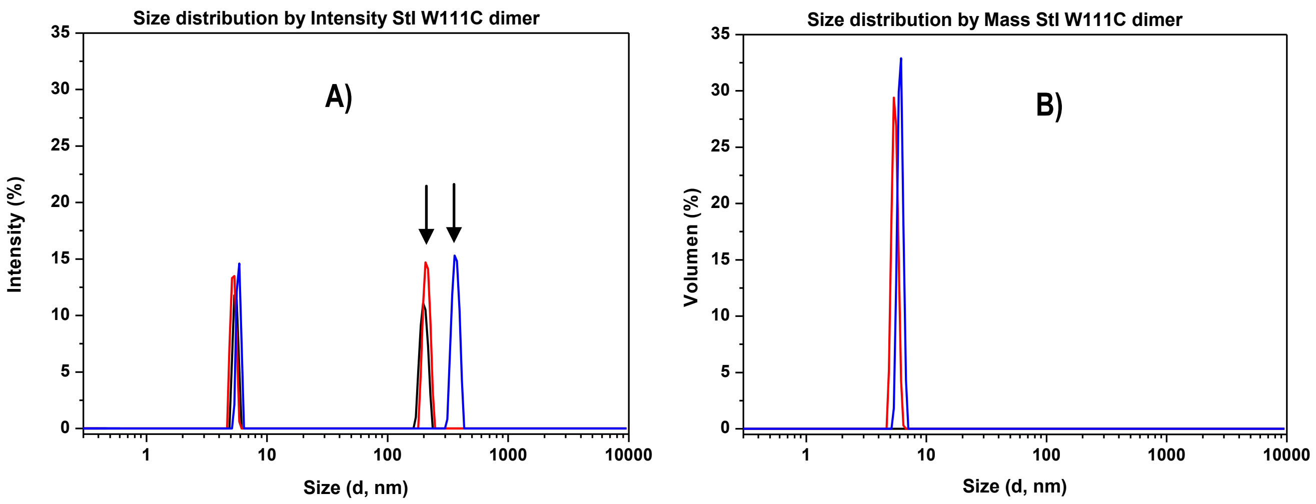

proteins	peaks	Mode ± SD (d, nm)	% Pd	Est. MW (kDa) (Mode ± SD)	% Intensity	% Mass	Peak Polydispersity
rStI	1	4.568 ± 0.9332	20.0	23.2 ± 4.7	85.9	100	Monodisperse
	2	115.5 ± 19.80	17.1	$4.46x10^4$ ± $8.45x10^3$	4.3	0.0	Monodisperse
	3	486.7 ± 92.24	19.0	$1.29x10^6$± $2.21x10^5$	9.8	0.0	Monodisperse
StI W111C dimer	1	5.878 ± 0.213	3.9	41.9 ± 2.7	36.2	100	Monodisperse
	2	359.2 ± 24.11	6.6	$6.33x10^5$ ± $2.57x10^4$	63.8	0.0	Monodisperse
StI W111C monomer	1	4.2 ± 0.9	19.0	19.7 ± 4.3	100	100	Monodisperse

Supplementary Fig. 5: The distributions of the intensity (A) and the volume (B) of dynamic light scattered by StI W111C dimer as a function of particle diameter. The distributions of the intensity (A) show large aggregates (indicated by arrows), but the distributions of the volume indicate that these signals are inconsiderable (0% mass). Only StI W111C dimer is identified according to 100% mass and monodispersity of the peak. The black, red, and blue traces indicate the distribution for three reading. Similar results for monomeric proteins were obtained for the StI W111C monomer and rStI, and the results are shown in the table below.

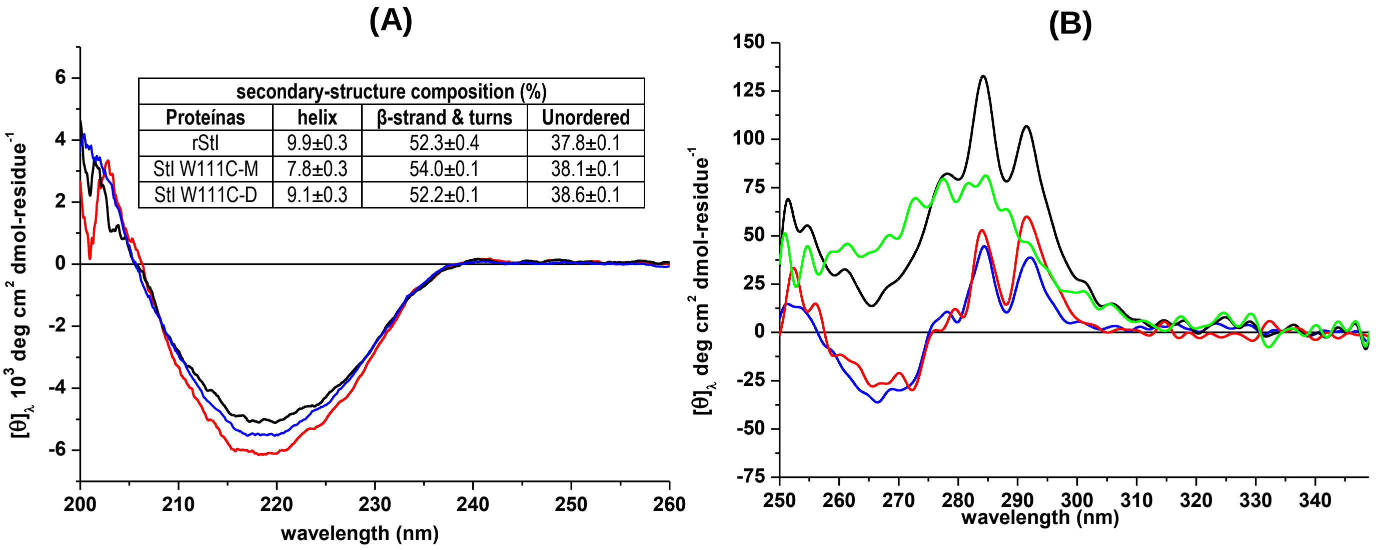

Supplementary Fig. 6: Conformational characterization by circular dichroism.

Far-UV **(A)** and Near-UV **(B)** CD spectra of StI W111C monomer and dimer forms, and rStI in TBS buffer. Protein concentration for Far-UV and Near-UV CD were 0.05 mg/mL and 0.3 mg/mL, respectively. In each case, the baselines have been subtracted by using control buffer not containing protein. StI W111C dimer (black line) and monomer (red line) forms, rStI (blue line), the Near-UV CD spectra difference (green line) between dimer and monomer corresponding to disulfide bridge contribution.

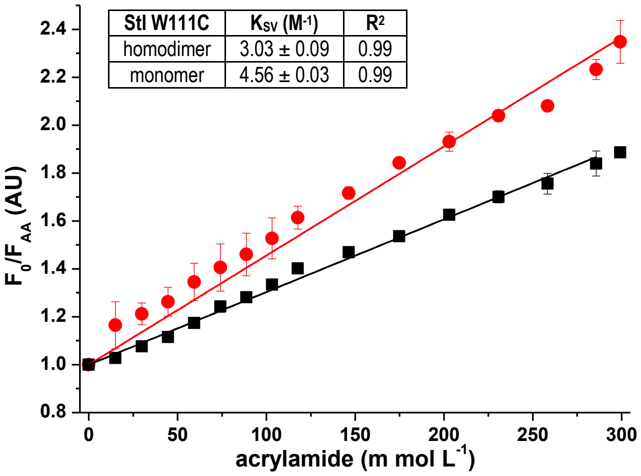

Supplementary Fig. 7: Stern-Volmer plots of acrylamide quenching of Trp fluorescence emission of StI W111C monomer and dimmer forms.

Stern-Volmer plots of tryptophan fluorescence intensity variation at 334 nm, after excitation at 295 nm, elicited by acrylamide addition. The fluorescence variation is expressed as the ratio between the fluorescence intensity at a given acrylamide concentration (F_{AA}) respect to the initial fluorescence without quencher addition (F_0). The following symbols represent the different protein samples: monomer (red solid circle) and dimer (black solid square) forms in solution.

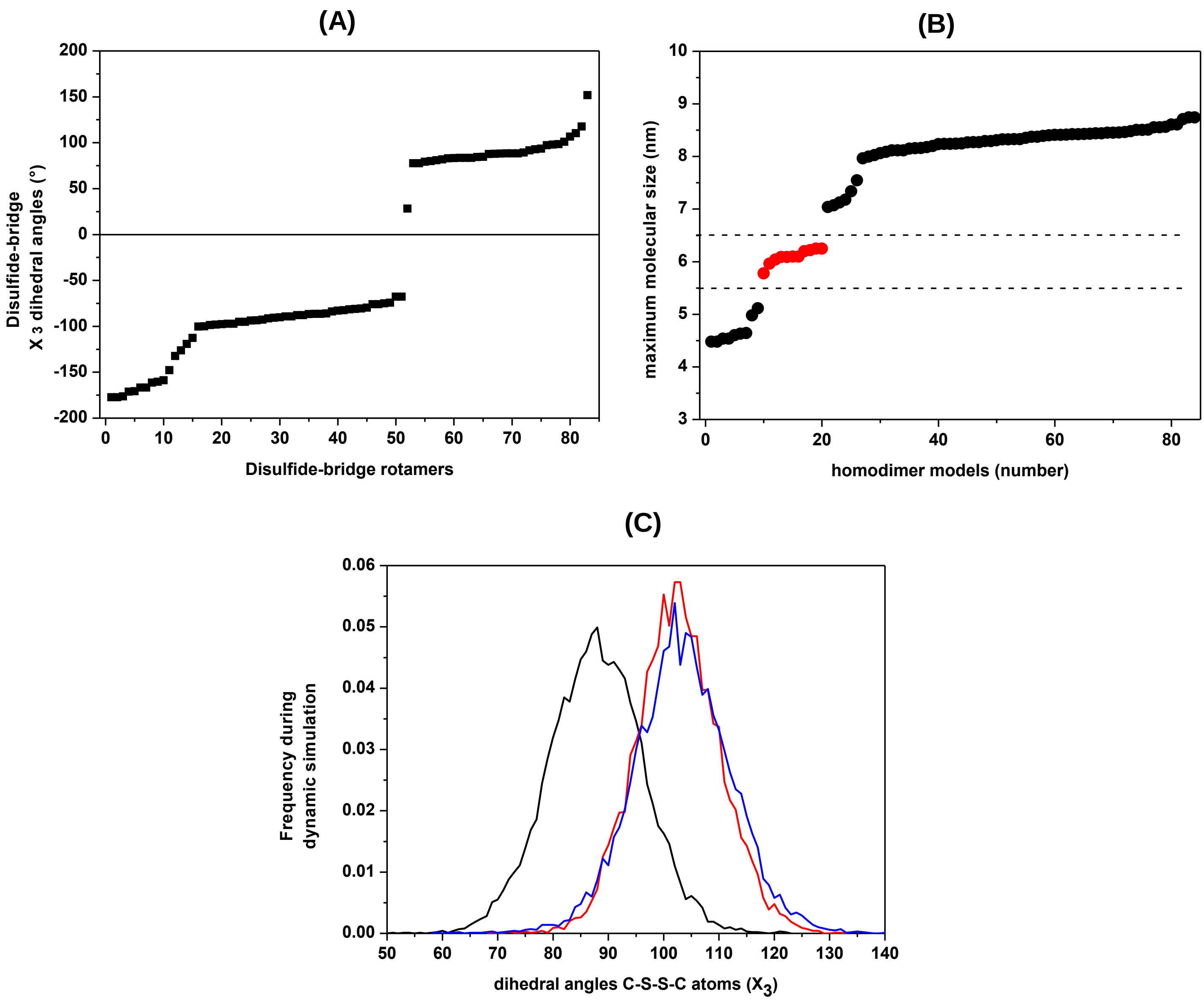

Supplementary Fig. 8: Dihedral angles of disulfide bridge and molecular sizes of homodimer.

Disulfide-bridge dihedral angles **(A)** and maximum molecular sizes **(B)** of 83 members rotamers library. Red points in panel B are the homodimer models with molecular size similar to DLS results. **(C)** Disulfide-bridge dihedral angles frequency distribution for three simulation dynamic model_14 (black line), model_25 (red line), and model_63 (blue line).

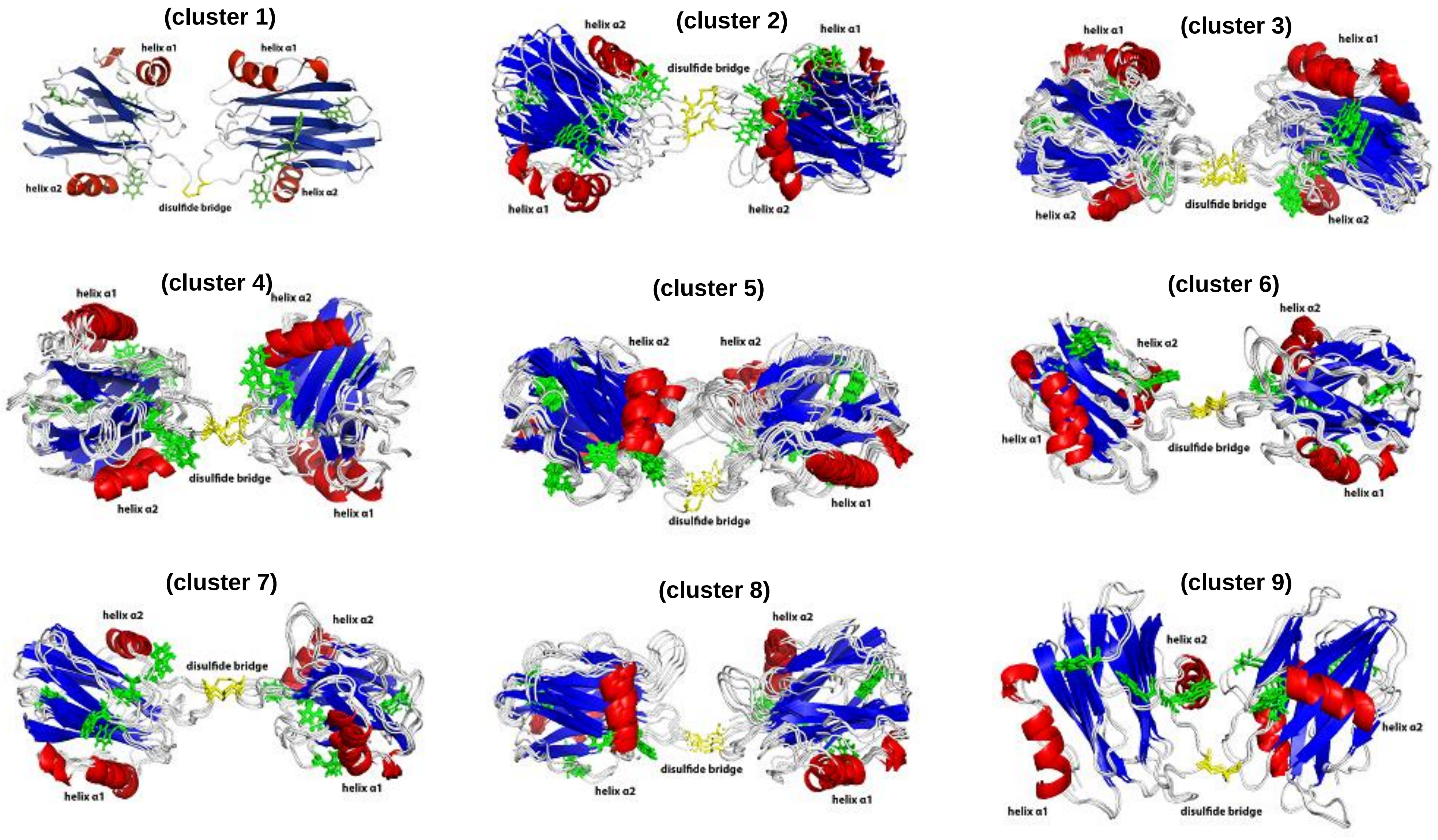

Supplementary Fig. 9: Clustering of the homodimer models.

Diagrams showing different aspects of the three-dimensional structure of clustered homodimers. The different elements of secondary structure are show in red (helixes), blue (sheets) and gray (loops and unordered structures). Tryptophan residues (green) and disulfide bridge (yellow) are also highlighted. The model composition of clusters is different: cluster 1 (one model), cluster 2 (four models), cluster 3 (eleven models), cluster 4 (nine models), cluster 5 (sixteen models), cluster 6 (twelve models), cluster 7 (five models), cluster 8 (seven models) and cluster 9 (two models). Images were produced with the *Pymol v1.7.6.6 softw*are for Windows [107].

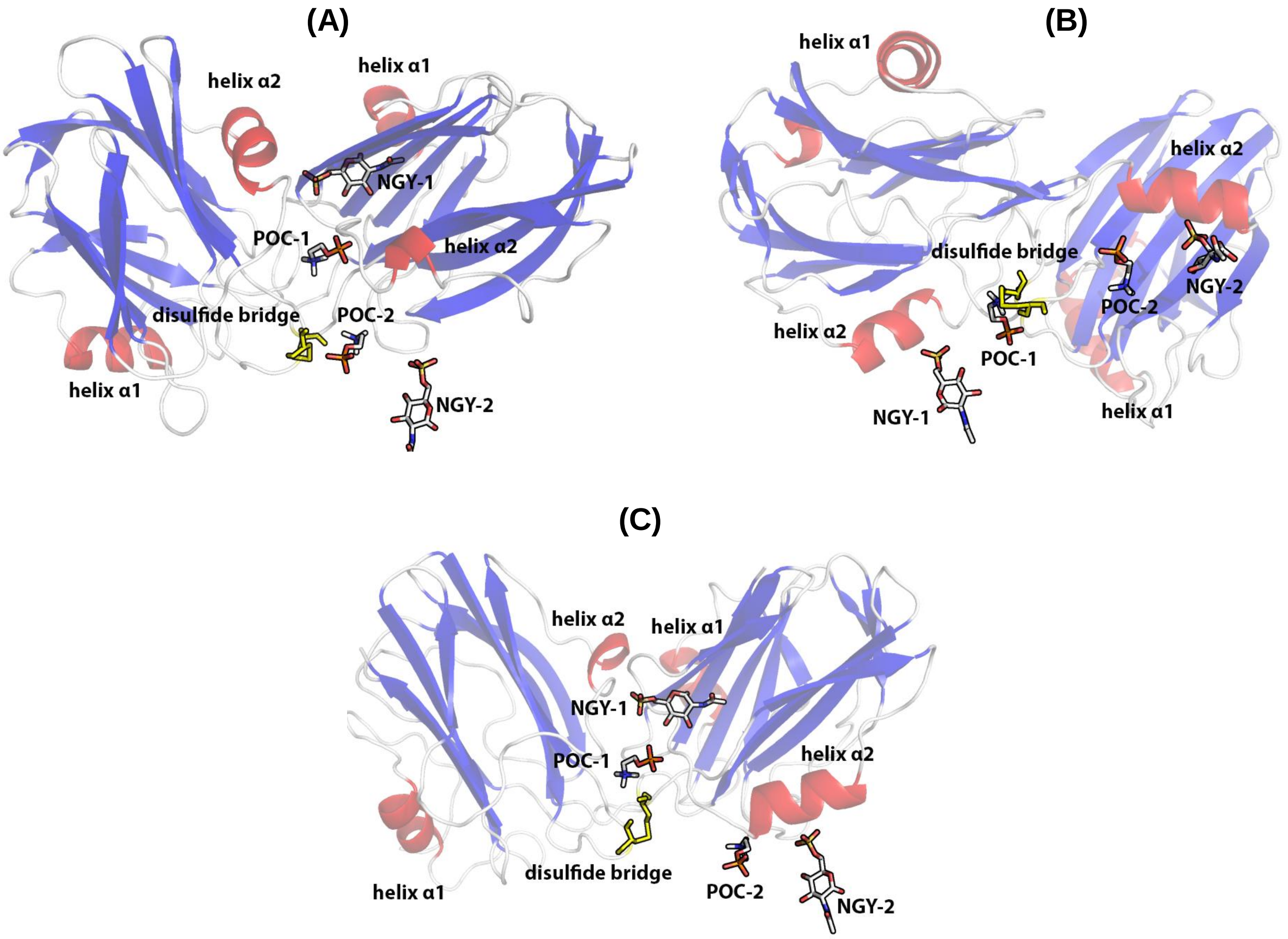

Supplementary Fig.10: Localization of the potential lipid and carbohydrate interaction sites in the structure of the dimer models.

All homodimer models were achieved after 100 ns of trajectory: **(A)** model_14 (31a_32b_1bsr), **(B)** model_25 (32a_31b_11ba), and **(C)** model_63 (90a_90b_1grg). The different elements of secondary structure are shown in red (helixes), blue (sheets) and gray (loops and unordered structures). The two phosphorylcholine (POC) [15] and N-acetyl glucosamine (NGY) [19] binding sites, and the disulfide bridge (yellow) in each dimer are also highlighted and labeled. Images were produced with the Pymol v1.7.6.6 software for Windows [107].

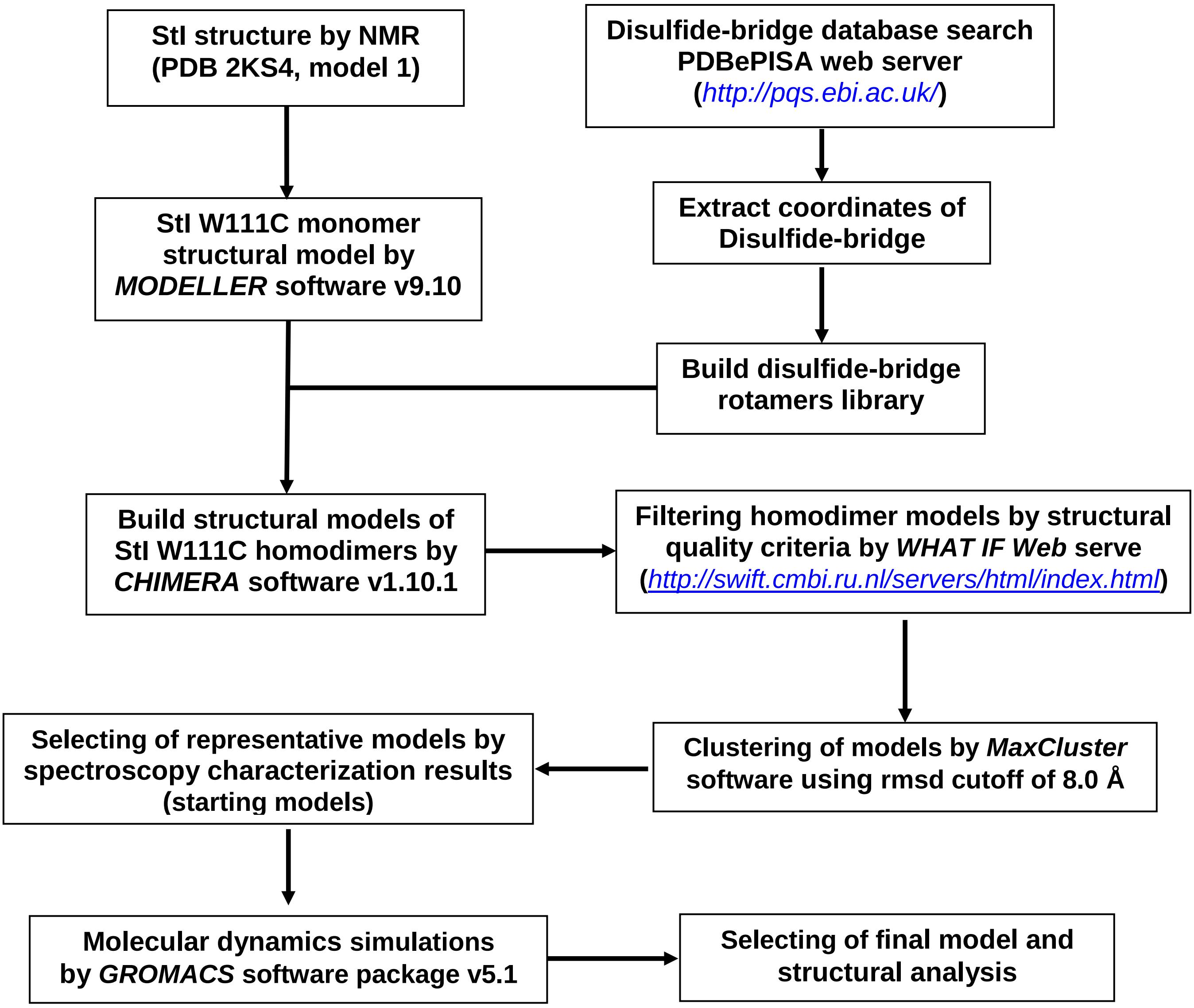

Supplementary Fig. 11: Scheme of the modeling of three-dimensional (3D) structure of StI W111C homodimers stabilized by disulphite bridge.

A 3D model of monomer StI W111C mutant was developed and used to predict homodimer models by a rigid-body global search using a disulfide bridge library. Models with good structural quality were clustered and representative models were employed as starting models for molecular dynamics simulations. A final model was selected by spectroscopy and structural criteria.

pdb id	num S-S	CYS-CYS residues	χ_3
2bmp	7	14A-79B<br>43A-111B<br>47A-104B<br>78A-78B<br>14B-79A<br>43B-111A<br>47B-113A	77.700<br>-75.900<br>-95.200<br>117.600<br>77.700<br>-75.900<br>-95.200
1pdg	4	43A-52B<br>52A-43B<br>43A-52B<br>52A-43B	-158.731<br>-161.401<br>-166.856<br>-166.856
2nsi	2	115A-115B<br>115C-115D	-126.292<br>-132.314
1qb3	2	90A-490C<br>290A-290B	-147.8<br>28.100
1bsr	2	31A-32B<br>32A-31B	92.723<br>89.179
1qi9	2	3A-41B<br>41A-3B	-89.366<br>-93.400
11bg	2	31A-32B<br>32A-31B	88.181<br>87.630
1qom	2	109A-109B<br>109B-109A	-97.623<br>151.789
1ccd	2	3A-69B<br>69A-3B	83.503<br>83.467
1utg	2	3A-69B<br>69A-3B	-67.730<br>-67.730
139l	2	68A-93B<br>93A-68B	-86.513<br>-86.521
1qmi	2	308A-308B<br>380C-308D	-112.786<br>-119.213
1aiu	1	73A-73B	98.526
1auc	1	73A-73B	97.334
1bmp	1	103A-103B	93.567
1gre	1	90A-90B	-177.400
1ert	1	73A-73B	97.864
1eru	1	73A-73B	100.920
1g8e	1	65A-65B	-74.324
1gra	1	90A-90B	-177.322
1grg	1	90A-90B	-171.300

pdb id	num S-S	CYS-CYS residues	χ_3
2vpf	25	26A-68A<br>26B-68B<br>26C-68C<br>26D-68D<br>26E-68E<br>26F-68F<br>26G-68G<br>26H-68H<br>51A-60B<br>51B-60A<br>51C-60D<br>60C-51D<br>51E-60F<br>51F-60E<br>51G-60H<br>51H-60G<br>57A-102A<br>61A-104A<br>57B-102B<br>61B-104B<br>57D-102D<br>61D-104D<br>57F-102F	84.300<br>84.600<br>81.800<br>88.400<br>83.500<br>83.100<br>80.900<br>83.000<br>-82.952<br>-82.400<br>-81.504<br>-80.643<br>-81.126<br>-86.700<br>-87.801<br>-87.801<br>-100.300<br>-90.300<br>-100.00<br>-91.300<br>-97.300<br>-92.800<br>-98.100
1vpf	4	51A-60B<br>60A-51B<br>51C-60D<br>60C-51D	-90.663<br>-85.899<br>-84.008<br>-89.231
2utg	2	3A-69B<br>69A-3B	-98.544<br>-79.724
11ba	2	31A-32B<br>32A-31B	88.423<br>79.959
1qq2	2	52A-173B<br>173A-52 B	110.426<br>106.528
1erw	1	73A-73B	91.528
1qou	1	145A-145B	-75.116
1sw6	1	404A-404B	-93.741
1tfg	1	77A-77B	79.308
1tgk	1	77A-77B	87.833
2sem	1	209A-209B	-97.182
2tgi	1	77A-77B	88.365
1grf	1	90A-90B	-170.680
1grs	1	90A-90B	-176.253
4gr1	1	90A-90B	-160.365

Supplementary Table 1. Disulfide bridge rotamer library.
Table shows the PDB identifier (pdb id), number of disulfide bridge on pdb (num S-S), residues and chains between are formed (CYS-CYS residues) and disulfide-bridge dihedral angles (X^3).
